# Helical Carbon Nanotubes Enhance the Early Immune Response and Inhibit Macrophage-Mediated Phagocytosis of *Pseudomonas aeruginosa*

**DOI:** 10.1371/journal.pone.0080283

**Published:** 2013-11-18

**Authors:** Brent E. Walling, Zhizhou Kuang, Yonghua Hao, David Estrada, Joshua D. Wood, Feifei Lian, Lou Ann Miller, Amish B. Shah, Jayme L. Jeffries, Richard T. Haasch, Joseph W. Lyding, Eric Pop, Gee W. Lau

**Affiliations:** 1 Department of Pathobiology, University of Illinois at Urbana-Champaign, Urbana, Illinois, United States of America; 2 Department of Electrical and Computer Engineering and Micro and Nanotechnology Laboratory, University of Illinois at Urbana-Champaign, Urbana, Illinois, United States of America; 3 Beckman Institute for Advanced Science and Technology, University of Illinois at Urbana-Champaign, Urbana, Illinois, United States of America; 4 Frederick Seitz Materials Research Laboratory, University of Illinois at Urbana-Champaign, Urbana, Illinois, United States of America; National Institute of Health (NIH), United States of America

## Abstract

Aerosolized or aspirated manufactured carbon nanotubes have been shown to be cytotoxic, cause pulmonary lesions, and demonstrate immunomodulatory properties. CD-1 mice were used to assess pulmonary toxicity of helical carbon nanotubes (HCNTs) and alterations of the immune response to subsequent infection by *Pseudomonas aeruginosa* in mice. HCNTs provoked a mild inflammatory response following either a single exposure or 2X/week for three weeks (multiple exposures) but were not significantly toxic. Administering HCNTs 2X/week for three weeks resulted in pulmonary lesions including granulomas and goblet cell hyperplasia. Mice exposed to HCNTs and subsequently infected by *P. aeruginosa* demonstrated an enhanced inflammatory response to *P. aeruginosa* and phagocytosis by alveolar macrophages was inhibited. However, clearance of *P. aeruginosa* was not affected. HCNT exposed mice depleted of neutrophils were more effective in clearing *P. aeruginosa* compared to neutrophil-depleted control mice, accompanied by an influx of macrophages. Depletion of systemic macrophages resulted in slightly inhibited bacterial clearance by HCNT treated mice. Our data indicate that pulmonary exposure to HCNTs results in lesions similar to those caused by other nanotubes and pre-exposure to HCNTs inhibit alveolar macrophage phagocytosis of *P. aeruginosa*. However, clearance was not affected as exposure to HCNTs primed the immune system for an enhanced inflammatory response to pulmonary infection consisting of an influx of neutrophils and macrophages.

## Introduction

Rapid advances in the synthesis of materials on a molecular scale has led to the production of a diverse range of nanomaterials including carbon nanotubes (CNTs). CNTs are rolled up sheets of graphite and can be designed to have a single wall (SWCNT), which are often 1-2 nanometers in diameter, or multiple walls (MWCNT) which can achieve external diameters from 10-250 nm. Although the diameters of nanotubes are very small, lengths of synthesized nanotubes can vary from nanometers to centimeters. CNTs have many distinctive physical and chemical properties due to their strong carbon-carbon (C-C) sigma bonds and sp2 hybridization, including high tensile strength, thermal and electrical conductivity [1]. With multiple potential industrial and medical applications, the demand for CNTs has risen rapidly, leading to mass production of nanotubes. However, with a size and shape similar to asbestos fibers, concern has been raised for pulmonary toxicity secondary to occupational and environmental exposure [2]. Much research has been devoted towards ascertaining the risk of CNT toxicity over the last 15 years [3,4].

In addition to direct pulmonary toxicity, investigators have questioned how inhalation of CNTs may also directly or indirectly alter pulmonary or systemic immunity. SWCNT exposure can activate alveolar macrophages, suggesting that nanotubes may enhance pulmonary immunity [5]. Additionally, Both SWCNTs and MWCNTs have been shown to exacerbate the allergic immune response in mice following ovalbumin sensitization [6,7]. However, others have shown that exposure to CNTs may suppress the immune response [8-11]. Extrapolating from those results, one could anticipate that exposure to CNTs would alter and inhibit the immune response to microorganisms. One study reported that SWCNT inhalation inhibited phagocytosis and decreased pulmonary clearance of *Listeria monocytogenes* in mice [12]. However, the same SWCNTs had no effect on the early immune response to *Toxoplasma gondii* infection [13] . Thus, consequences of CNT-induced alterations of the host immune response to pathogens are not universal and studies focusing on pulmonary pathogens are needed. To date, there has been no published research as to whether or how CNT exposure alters the immune response to known pulmonary pathogens.

*Pseudomonas aeruginosa* is an opportunistic, ubiquitous, Gram-negative pathogen and a major cause of nosocomial pulmonary infections [14]. Pulmonary infection in immune-competent individuals is usually self-limiting, utilizing a robust acute inflammatory response to clear the infection and prevent colonization. However, *P. aeruginosa* is a significant cause of chronic infection and morbidity in patients with cystic fibrosis and chronic obstructive pulmonary disease, ventilator-associated pneumonia, and immunocompromised patients. Given the concern for pulmonary exposure to CNTs, the various immunomodulatory properties of CNT reported in the literature, and the lack of CNT toxicity research using pulmonary pathogens, our lab investigated the effect helical carbon nanotube (HCNT) exposure had on subsequent pulmonary infection of *P. aeruginosa* in mice. 

## Materials and Methods

### Chemicals and cell lines

Chemicals were purchased from Sigma-Aldrich, unless stated otherwise. 

### Preparation of HCNTs

HCNTs (Cheap Tubes Inc.) were suspended to 1 mg/ml in DMEM without phenol (Gibco) with dispersal media (0.01% Tween-80 in phosphate buffered saline (PBS)), vortexed, sonicated on ice, and diluted to desired concentrations. The enotoxin concentration was determined by the *Limulus* amebocyte assay (ToxinSensor Chromogenic LAL Endotoxin Assay Kit, GenScript, Piscataway, NJ, detection limit 0.005 EU/ml). Dispersion following sonication was determined by measuring light absorption at 550 nm. Physical characteristics of the starting material were assessed with transmission and scanning electron microscopy (TEM, SEM), and Raman spectroscopy. Elemental analysis was performed using energy dispersive X-ray spectroscopy (EDX) and X-ray photoelectron spectroscopy (XPS). The size distribution and zeta potential of dispersed particles in the dispersal media was performed using a Zetasizer Nano ZS(Red Badge) ZEN3600 (Malvern Instruments, Paris, France) (see Text S1 and Figures S1-S7). 

### Molecular, cytotoxicity, and immunological assays

Protein concentrations were determined by the BCA protein assay kit (Pierce). Lactate dehydrogenase levels were determined by the CytoTox 96 non-radioactive cytotoxicity assay (Promega). KC and MCP-1 concentrations were determined using ELISA kits (R&D Systems).

### Ethical statement

The animal study was carried out in strict accordance with the recommendations in the Guide for the Care and Use of Laboratory Animals of the National Institutes of Health. The protocol was approved by the Institutional Animal Care and Use Committee (IACUC) at the University of Illinois at Urbana-Champaign (Protocol Number: 10234).

### HCNT exposure

Animal studies were performed with approval by the Institutional Animal Care and Use Committee at the University of Illinois at Urbana-Champaign. Six-week old wild-type CD-1 mice (Charles River Laboratories) were housed in positively ventilated microisolator cages with automatic recirculating water, located in a room with laminar, high efficiency particle accumulation-filtered air. The animals received autoclaved food, water, and bedding. Exposure to HCNTs was performed by intranasally inoculating mice with 50 ug of HCNTs in 50 µl of dispersal media or equivalent volume of dispersal media alone either once or twice/week for 3 weeks (Monday and Thursday). Mouse lungs were lavaged or collected for histopathology 24 hours after the last treatment. 

### Pulmonary clearance of *P. aeruginosa*

Pulmonary clearance of *P. aeruginosa* was assessed following 3 weeks of exposure to HCNTs as described above by giving mice a single intranasal dose of PAO1 (10^7^ CFU in 50 µl) at 6 or 72 hours following the last HCNT administration[12]. After 24 hours, mouse lungs were harvested for bacterial enumeration, histopathology, or lavaged for immune cell enumeration, and chemokine determination. Depletion of neutrophils was accomplished by intraperitoneal (IP) injection of 0.2 mg anti-Ly6G antibody (BioXCell) 48 hours following the last HCNT administration and infecting mice with 10^4^ CFU PAO1 24 hours later. Depletion of systemic macrophages was accomplished by IP injections of clodronate liposomes (clodronate liposomes.com) at 72 hours (5.0 mg/mouse) and 24 hours (1.0 mg) prior to infection. Neutrophil and macrophage depletions were assessed by analyzing the bronchoalveolar lavage (BAL) samples and blood smears taken from abdominal venipuncture (n=3-4).

### Assessment of phagocytosis by alveolar macrophages

Phagocytosis of *P. aeruginosa* by alveolar macrophages following HCNT exposure was accomplished by giving mice a single intranasal dose of a PAO1 strain expressing green fluorescent protein (PAO1-GFP) (10^7^ CFU in 50 µl). After 1 hour, mouse lungs were lavaged for cell collection and phagocytosis determination. 

### Bronchoalveolar lavage

BAL was performed as previously described [15,16]. Leukocytes were enumerated by a hemocytometer. Cell differential was determined microscopically following cytospin preparation of cells stained with Kwik-Diff (Thermoscientific). At least 200 cells per slide were counted. Cells collected for confocal microscopy were concentrated on glass slides by cytospin and fixed fixed with 4% paraformaldehyde for 1 hour. Phagocytosis of PAO1-GFP was determined by evaluating at least 200 macrophages by confocal microscopy. 

### Histopathology evaluation of mouse lungs

Mouse lungs were collected for histopathological analyses as described previously [17]. Lung sections were stained with hematoxylin and eosin (H&E) or with Alcian blue.

### Statistical analysis

Normality of the data was evaluated using the Anderson-Darling normality test with rejection of normality when p-value < 0.05. Data were then analyzed for statistical significance by Student's t-tests, with differences between means considered significant when p-value < 0.05. For comparing the means of groups of three or more, data were analyzed for statistical significance by ANOVA followed by Tukey’s tests for comparison between the means.

## Results

### Characterization and dispersal of HCNTs

**Figure 1:**
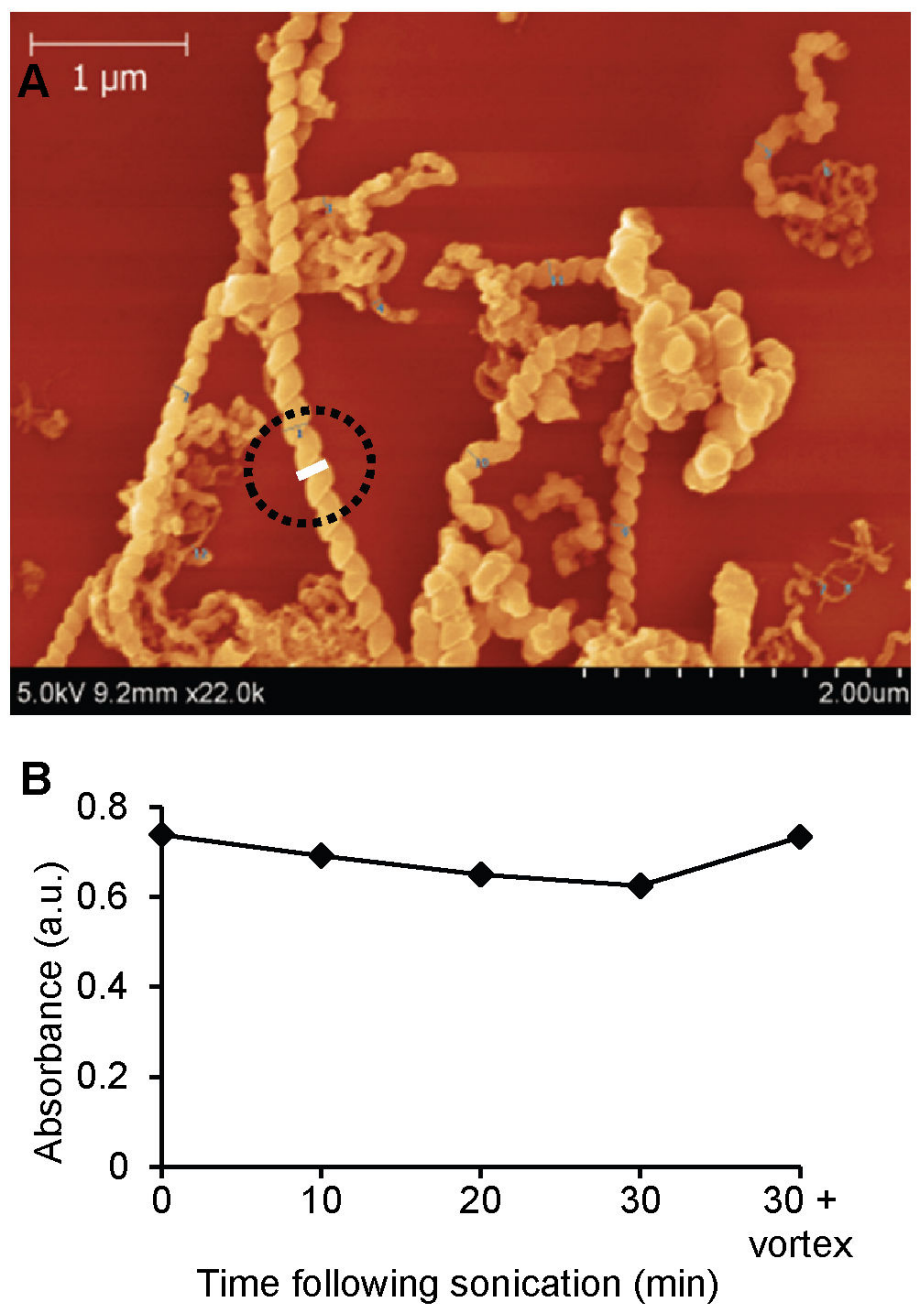
HCNTs resuspended in 0.01% Tween-80 are adequately dispersed prior to use. (**A**) A representative SEM image of HCNTs shows moderate variability in the diameter of individual tubes as well as sharp kinks and folding. The circled area highlights the diameter measurement using Gwyddion software (see supporting information). (**B**) Absorbance as a measurement of dispersion following 5 minutes of sonication. The decrease in absorbance over time is from dispersed HCNTs settling by gravity. A brief vortexing resuspends the HCNTs.

HCNTs (Cheaptubes, Inc. Brattleboro, VT) were chosen for our study due to their unique shape in comparison to other CNTs. Scanning electron microscopy (SEM) images show many of the tubes folding into complex structures and/or having sharp kinks (Figure 1A). A summary of their physical characteristics is shown in Table 1. SEM of the dispersed HCNTs demonstrated a diameter distribution of 50-500 nm, with an average about ~200 nm. The length distribution was determined by edge extraction from SEM images with NeuronJ [18], finding a range of lengths from 1.1-2.7 µm with average ~1.9 µm [19]. Using the diameter and length information, the specific surface area (SSA) of the HCNTs was calculated to be 38.6 ± 28.7 m^2^/g (see Text S1). X-ray photoelectron spectroscopy (XPS) revealed that HCNTs are comprised of 99.5% carbon, 0.43% aluminum, and 0.08% chlorine. Oxygen was also present and likely due to adsorbed water. Energy dispersive X-ray spectroscopy (EDX) revealed predominantly carbon and trace amounts of iron and oxygen. Because iron was not detected by XPS, it comprised < 0.01% of the HCNTs. Particle size distribution of dispersed samples demonstrated a mean size of 532 nm. The zeta potential of dispersed HCNTs in dispersal media was determined to be -3.04 mV. Full details of the characterization of HCNTs can be found in the Figures S1-S7. Dispersal using 0.01% Tween-80 in PBS, which has been used with no noticeable biologic effects at low concentrations [6,20], followed by sonication achieved satisfactory dispersal with minimal agglomeration (Figure 1B). Endotoxin levels of HCNTs in dispersal media were < 0.005 EU/ml.

**Table 1:** Characteristics of HCNTs.

Width	µ = 199.9 ± 127.8 nm
Length	µ = 1.9 ± 0.8 µm
Surface area	38.6 ± 28.7 (m^2^/g)
Purity	> 99.5% carbon

### The inflammatory response to HCNTs in vivo

**Figure 2:**
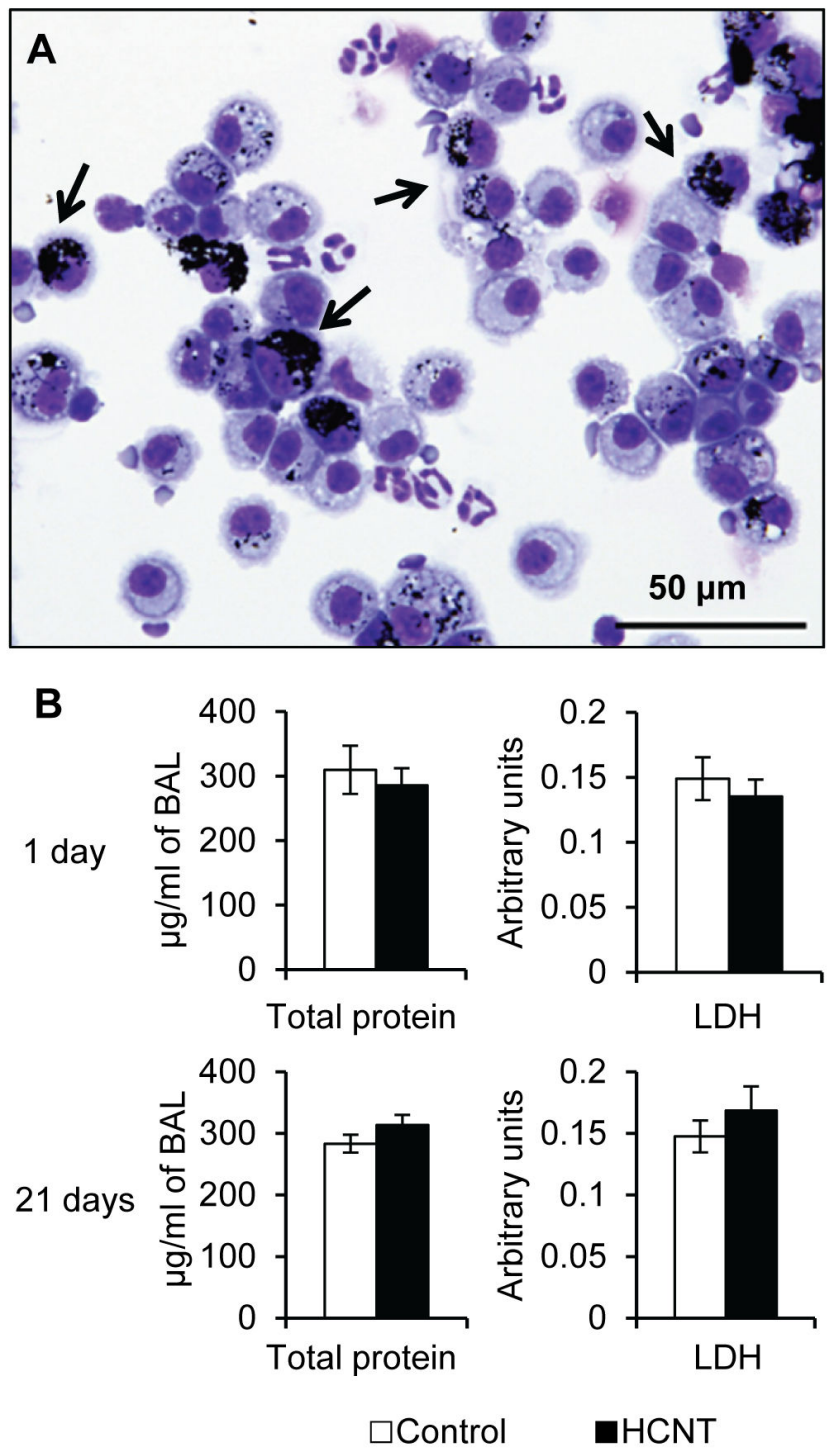
Acute and repeated exposure to HCNTs result in phagocytosis of HCNTs by alveolar macrophages. (**A**) Cytospin preparation of BALF from an HCNT treated mouse 24 hours after one administration. Many of the macrophages have phagocytized HCNTs (arrows). (**B**) Total protein and LDH levels measured from the BALF of mouse lungs acutely (1 day) and repeat (21 days) exposed to HCNTs. Black bars = HCNT, white bars = control. n = 3-4 for each group. * p < 0.05. Error bars indicate standard error of the mean.

**Figure 3:**
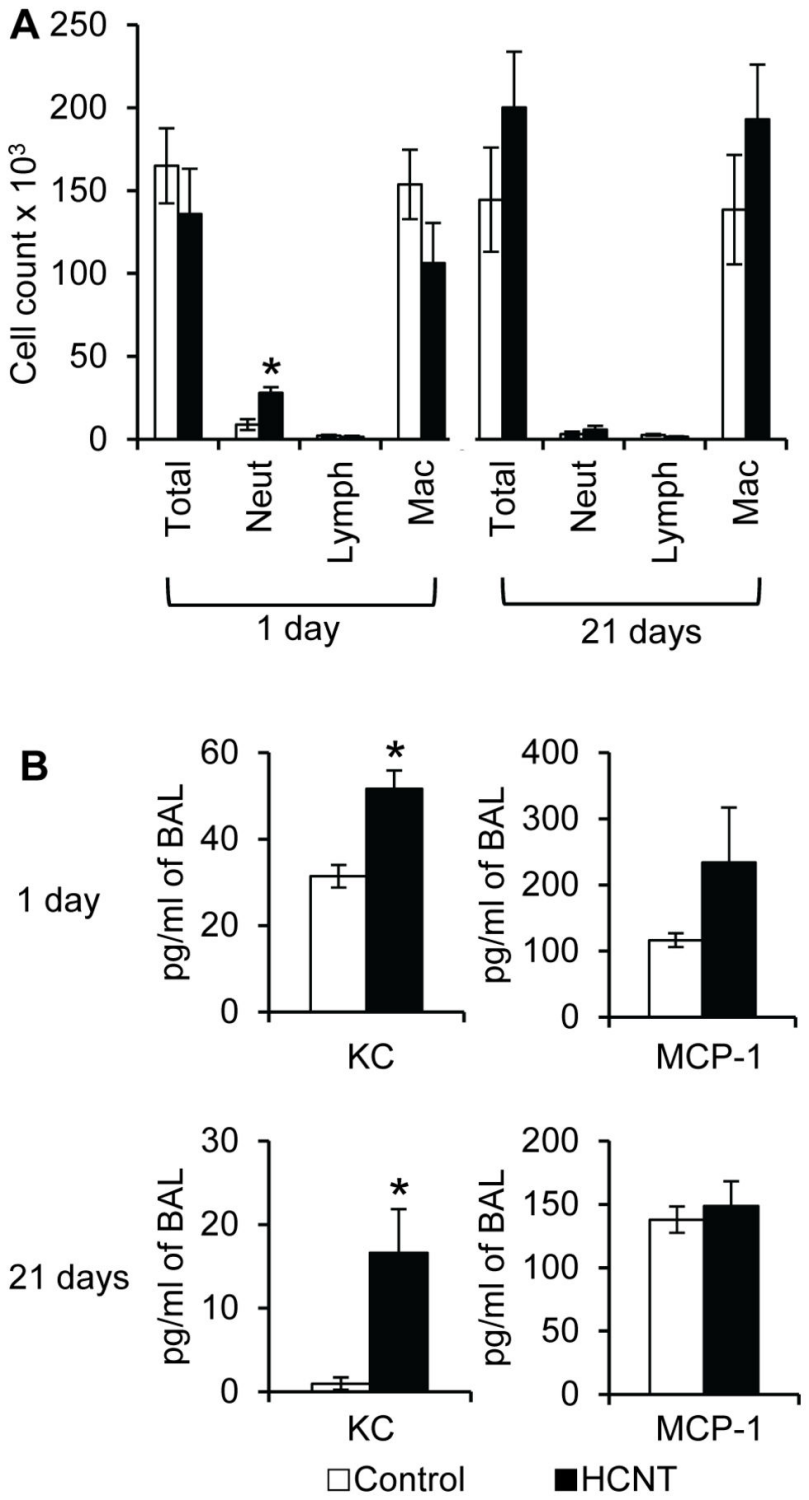
Acute and repeat exposure to HCNTs result in changes in the murine pulmonary leukocyte population. (**A**) Quantitative analyses of the leukocyte populations in the BALF 1 day and 21 days after exposure to HCNTs. (**B**) KC and MCP-1 measured from the BALF of mouse lungs acutely (1 day) and repeatedly (21 days) exposed to HCNTs. KC levels were significantly elevated in the BAL following acute and repeated exposure to HCNTs.. Black bars = HCNT, white bars = control. n = 3-4 for each group. * p < 0.05. Error bars indicate standard error of the mean.

Without any information regarding HCNT toxicity in the literature, we first exposed CD-1 mice to 50 μg of HCNTs either once or 2X/week for 3 weeks (repeat exposure). Macrophages readily phagocytized HCNTs (Figure 2A, arrows). Cytological analyses from BAL analyses showed that 71% and 88% of the macrophages in the once and repeat exposure mice, respectively, had phagocytized HCNTs (Figure S8). HCNTs do not appear to be significantly cytotoxic as both total protein and lactate dehydrogenase levels in the BAL fluid (BALF) were not statistically different between the HCNT and control groups following single or repeat exposures (Figure 2B). HCNT exposure resulted in a statistically significant increase in the number of neutrophils in the BALF 24 hours after a single inoculum of HCNTs was administered and macrophages were elevated in mice repeated exposed to HCNTs (Figure 3A). Mouse neutrophil chemotactic chemokine KC was significantly elevated in the BALF of single (64%) and repeat (1593%) HCNT exposed mice compared to controls. The levels of macrophage chemotactic chemokine MCP-1 were not significantly different (Figure 3B).

### Repeated exposure to HCNTs induces pulmonary lesions

**Figure 4:**
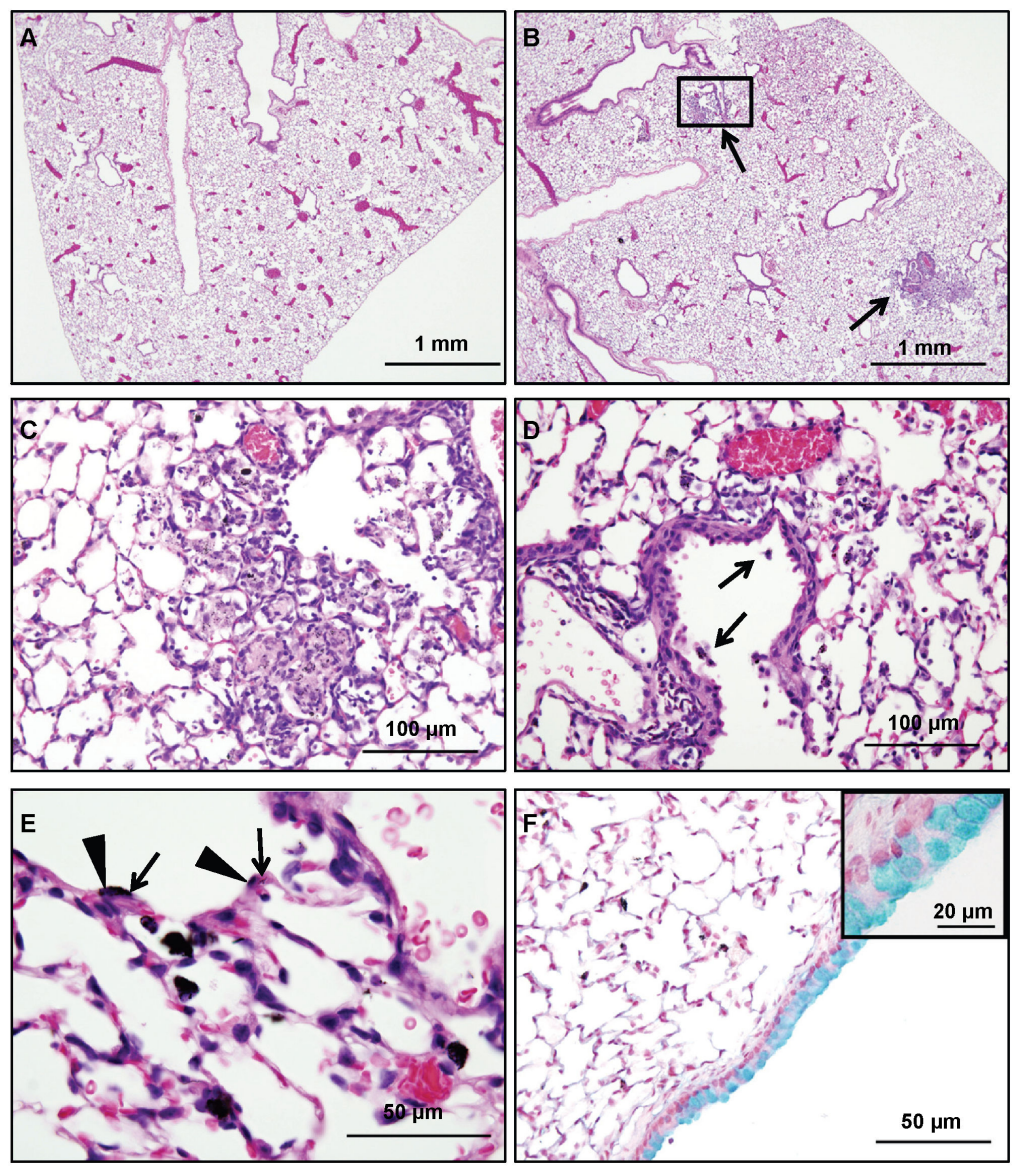
Repeated exposure to HCNTs results in multiple pulmonary lesions. (**A**) Mice exposed to the dispersal media (PBS + 0.01% Tween-80) showed no significant changes in the bronchi or alveolar spaces. (**B**) Mice exposed to HCNTs developed multiple granulomas (arrows). (**C**) A higher magnification of the rectangular inset from B. (**D**) HCNT laden macrophages can be found both within the terminal bronchiole (arrows) and alveolar spaces. (**E**) HCNTs can be found within the alveolar walls and epithelial cells (arrows) and can be distinguished from epithelial cell nuclei (arrowheads). (**F**) Alcian blue staining of the lungs repeatedly exposed to HCNTs show evidence of goblet cell hyperplasia in the larger conducting airways. Inset is a higher magnification of the bronchiolar epithelium.

Repeated exposure to CNTs has resulted in a variety of pulmonary lesions in animal models including granuloma formation, bronchiolar and alveolar fibrosis, and goblet cell hyperplasia [21,22]. In our models, the lungs from control mice receiving dispersal media were unremarkable (Figure 4A). In contrast, repeated administration of HCNTs resulted in multiple granulomas (Figures 4B and C) in the lungs of mice. HCNT-laden macrophages could be found in all lung lobes examined within the terminal bronchioles and alveolar spaces (Figures 4D and E). The lungs also contained foci of goblet cell metaplasia, a phenomenon reported in other studies of CNT exposure [6,25,26], confined to the proximal airways (Figure 4F). 

### Pulmonary immune response to *P. aeuruginosa* following repeated HCNT administration

**Figure 5:**
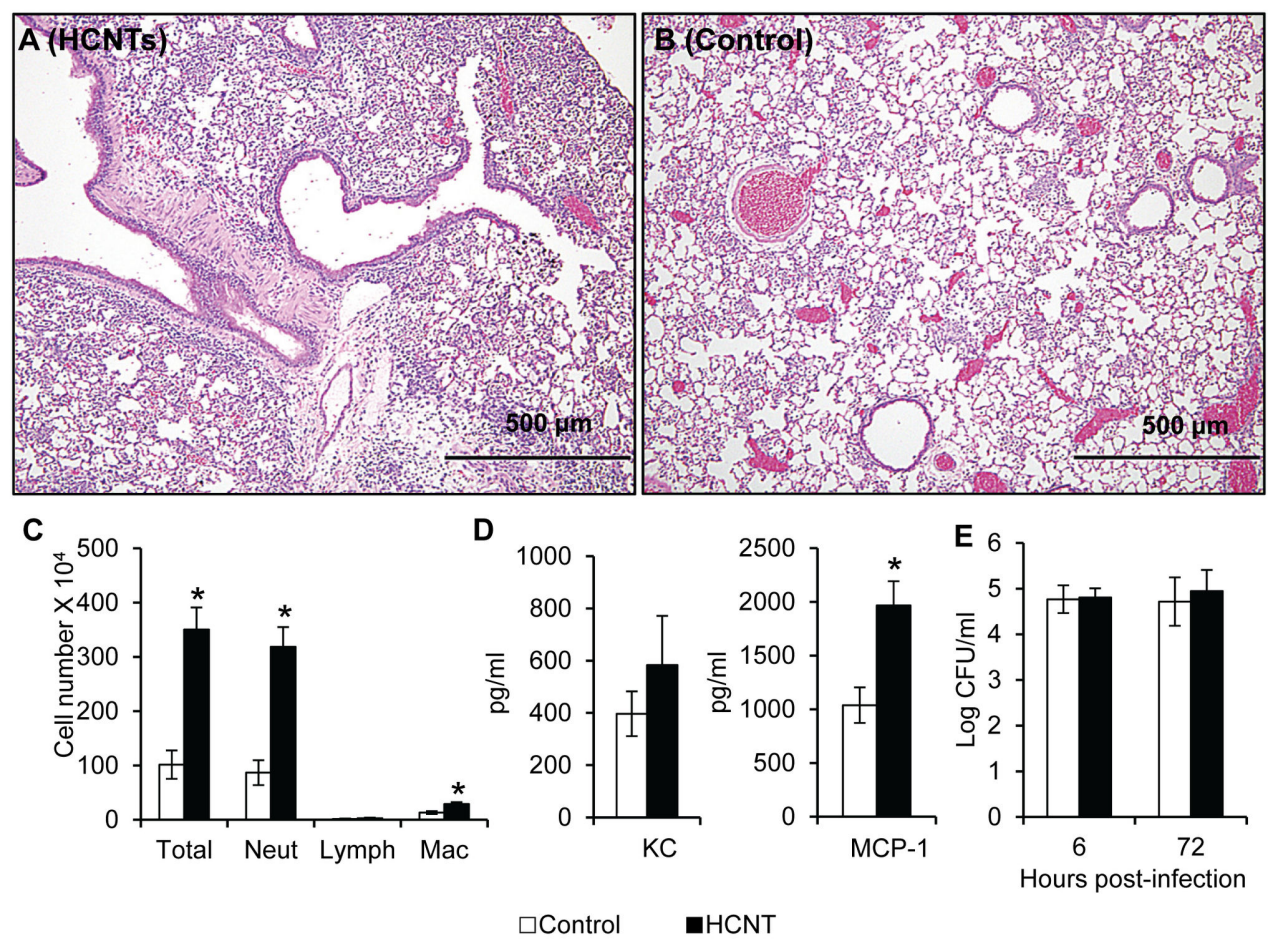
Repeated HCNT exposure enhances pulmonary inflammation without impeding clearance of *P. aeruginosa*. Mice received HCNTs or PBS/Tween 80 twice/week for 3 weeks. Three days after the last treatment, *P. aeruginosa* strain PAO1 (10^7^/mouse) was given to all mice. (**A**-**B**) Representative images of paraffin embedded lungs from mice 24 hours after PAO1 was administered show that HCNT treated mice had a more robust inflammatory response (A) compared to control mice (B). (**C**) Cytospin preparations from the BALF of mice 24 hours after PAO1 administration shows a significant increase in the numbers of neutrophils and macrophages from HCNT treated mice compared to controls. (**D**) KC and MCP-1 measured from the BALF of mouse lungs after PAO1 infection following repeated exposure to HCNTs or dispersal media. All parameters were elevated in HCNT exposed mice compared to controls, with MCP-1 reaching statistical significance. (**E**) Clearance of PAO1 in mice was determined by homogenization of whole lungs followed by serial dilutions and plating. Mice were infected 6-72 hours after the last HCNT treatment. Repeated administration of HCNTs did not affect the clearance of PAO1 from the lungs 24 hours after PAO1 infection. Black bars = HCNT, white bars = control. n = 5 to 6 mice for each group. * p < 0.05. Error bars indicate standard error of the mean.

To examine whether repeated HCNT exposure could inhibit clearance of *P. aeruginosa* strain PAO1, we modified a protocol from Shvedova et al. [12]. A cohort of CD-1 mice was repeatedly exposed to HCNTs as above. In order to minimize the influence of free HCNTs interacting with pathogens in the alveolar spaces, mice were given PAO1 (10^7^ CFU/mouse) 72 hours after the last HCNT administration. Mice lungs were analyzed 24 hours later. Histologic examination of the lungs showed a more robust inflammatory response to PAO1 infection in the HCNT exposed mice (Figure 5A) than the dispersal medium controls (Figure 5B). Greater number of leukocytes, neutrophils, and macrophages were recovered from the BALF of HCNT exposed and infected mice when compared to infected mice that only received the dispersal media (Figure 5C). Neutrophil and macrophage chemotactic factors KC and MCP-1 were elevated by 47%, and 89%, respectively, in the BALF of HCNT exposed mice compared to controls following PAO1 infection (Figure 5D), which supported the histologic evidence of enhanced leukocyte trafficking. Interestingly, there was no significant difference in the clearance of PAO1 from HCNT exposed mice compared to control mice (Figure 5E). We repeated the experiment and infected the mice 6 hours after the last HCNT treatment to minimize egress of HCNT-laden macrophages. Again, there was no significant difference in the clearance of PAO1 from HCNT compared to control mice (Figure 5E).

**Figure 6:**
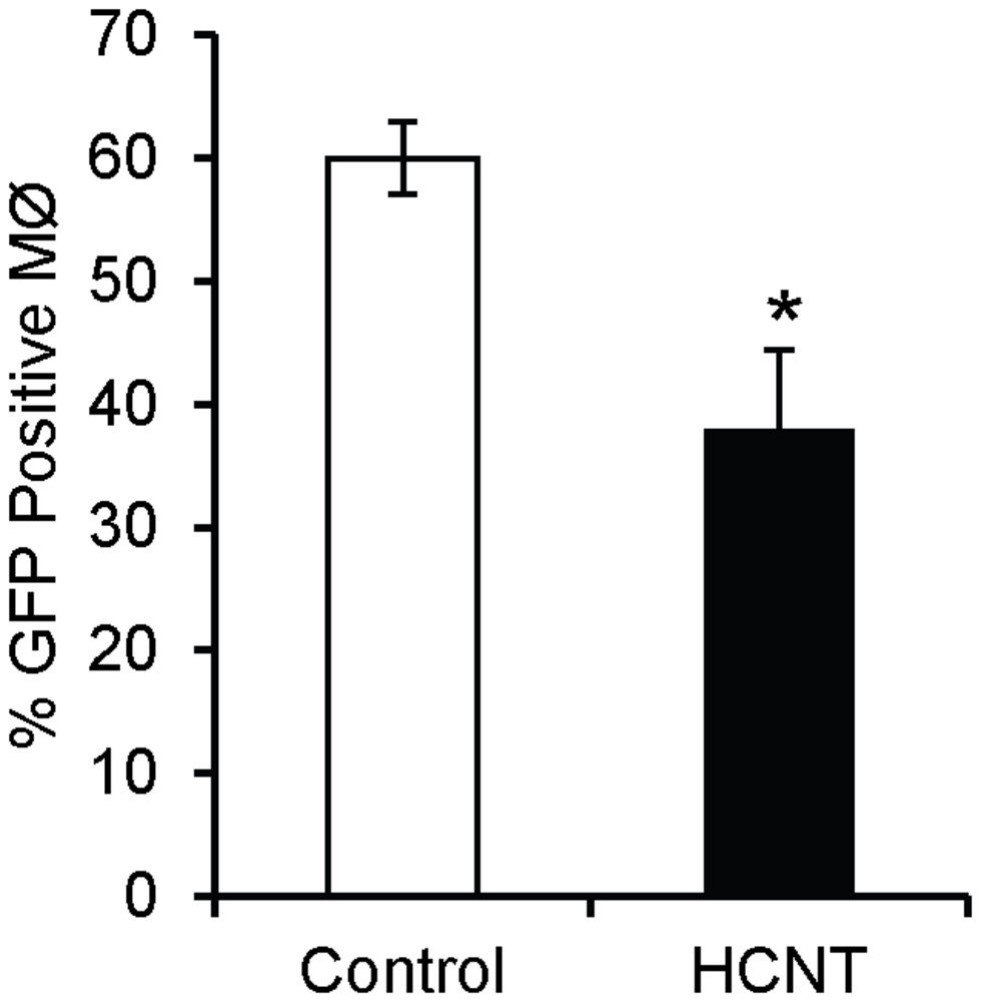
HCNTs inhibit phagocytosis of *P. aeruginosa*. Macrophages from mouse lungs pre-exposed to HCNTs are less efficient at phagocytizing PAO1-GFP. n = 3 mice for each group, a minimum of 200 macrophages were counted per mouse. Black bars = HCNT, white bars = control. * p < 0.05. Error bars indicate standard error of the mean.

Because clearance of pathogens relies on multiple factors, including neutrophils and macrophages, and prior work has shown direct evidence of inhibition of bacterial phagocytosis by CNTs, we repeated the three week exposure protocol, infected mice with 10^7^ CFU PAO1 expressing GFP, and recovered the alveolar macrophages for confocal microscopy. Analysis showed that a significantly greater percentage of alveolar macrophages from control mice had phagocytized bacteria compared to HCNT treated mice (Figure 6).

### Depletion of select lineages of leukocytes alters the clearance of *P. aeruginosa* in HCNT exposed mice

**Figure 7:**
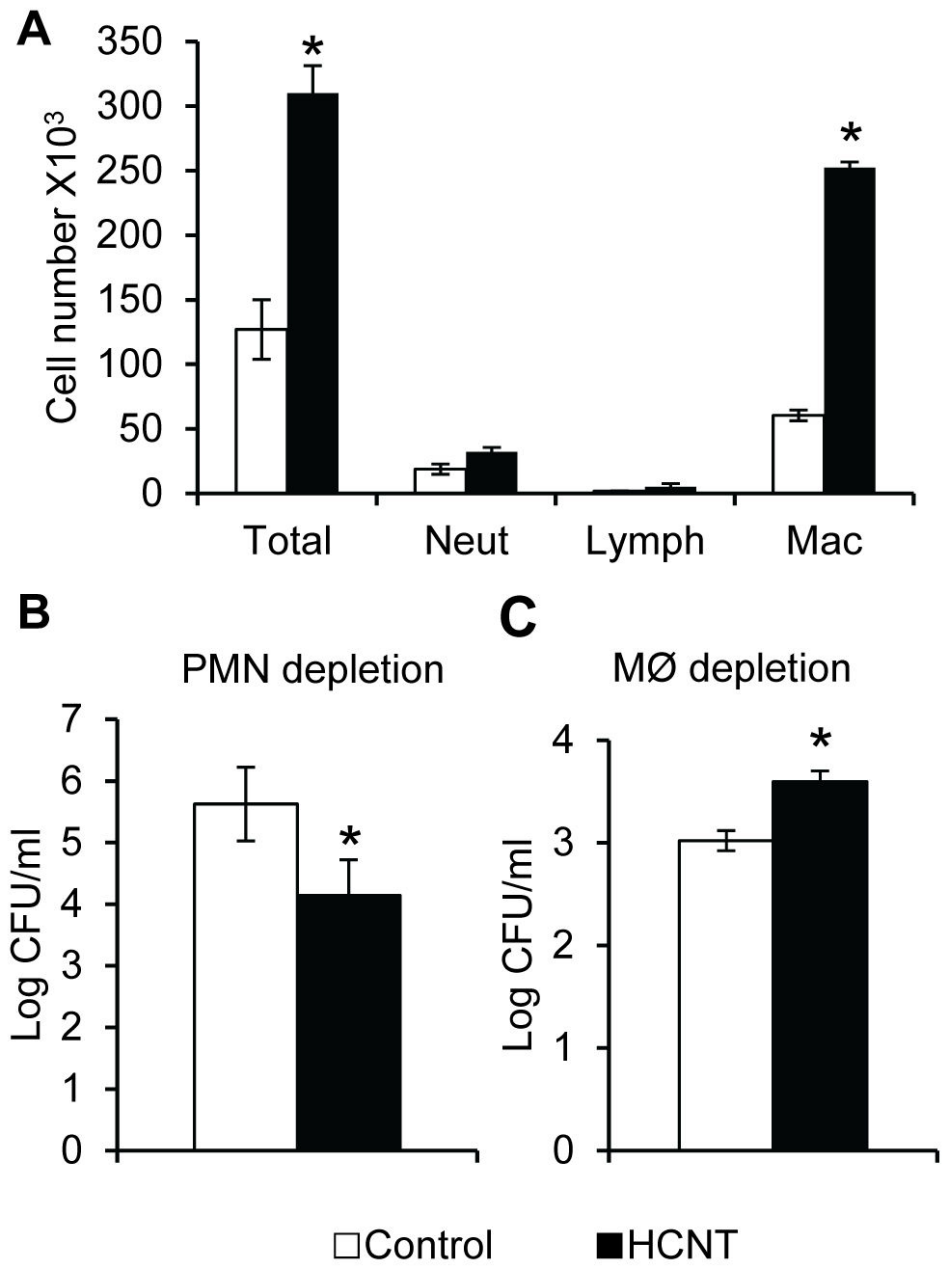
HCNT pre-exposure affects clearance of *P. aeruginosa* in mice depleted of specific lineages of leukocytes. (**A**) Preexposure to HCNTs in neutrophil depleted mice significantly increased the number of macrophages in the BAL in response to PAO1 infection. n = 5 mice for each group. * p < 0.05. Error bars indicate standard error of the mean. (B) HCNT exposed mice had increased clearance of PAO1 from the lungs than control mice when depleted of neutrophils prior to infection. (C) HCNT exposed mice had reduced clearance of PAO1 when systemically depleted of macrophages. Black bars = HCNT, white bars = control. n = 8 to 11 mice for each group. * p < 0.05. Error bars indicate standard error of the mean.

To clarify the roles of neutrophils and macrophages during the immune response to *P. aeruginosa* infection in HCNT-exposed lungs, we used either clodronate liposomes to deplete macrophages or anti-Ly6G antibodies to deplete neutrophils. Clodronate and Ly6G antibody treatment significantly alters the leukocyte differentials in both the BAL and in circulation following PAO1 infection (Table 2). Mice treated with anti-Ly6G antibodies had significantly decreased numbers of neutrophils in both the BAL and circulation compared to non-depleted mice. Surprisingly, HCNT exposed mice depleted of neutrophils prior to PAO1 infection had a significantly increase in the number of macrophages in the BAL and decreased PAO1 recovered from the lungs by 1.5 log compared to control mice (Figure 7A, B). On the other hand, HCNT exposed mice which were systemically depleted of macrophages were less able to clear a PAO1 infection than control mice by about 0.5 log (Figure 7C).

**Table 2:** Leukocyte differentials from the blood and BAL of mice after infection by PAO1.

	Treatment	% Neutrophils	% Lymphocytes	% Macrophages
	No pretreatment	73.2 ± 2.5	2.2 ± 0.7	24.7 ± 2.4
BAL	Clodronate liposomes	86.7 ± 1.1*	0.7 ± 0.2	12.7 ± 0.9
	Anti Ly6G antibody	23.2 ± 4.8*^†^	2.3 ± 0.3^†^	74.5 ± 5.0*^†^
	No pretreatment	44.3 ± 5.6	34.3 ± 6.5	21.5 ± 2.3
Blood	Clodronate liposomes	56.5 ± 3.8	33.2 ± 2.8	10.3 ± 1.7*
	Anti Ly6G antibody	2.5 ± 0.8*^†^	81.2 ± 3.7*^†^	16.3 ± 3.5

Values are means ± SE. n = 3 mice per group. * p < 0.05 vs. no pretreatment. ^†^ p < 0.05 vs. Clodronate liposomes.

## Discussion

In this study, we analyzed the pathogenesis of acute and repeated pulmonary exposure to HCNTs and their impact on the pulmonary immune response and clearance of *P. aeruginosa*. Repeated exposure to HCNTs produced lesions in mice similar to what has been reported following exposure to SWCNTs and MWCNTs including granulomas [23-25] and goblet cell hyperplasia ([25-27] as well as elevated proinflammatory cytokines [24,26]. Furthermore, our results have shown that HCNT exposure can inhibit phagocytosis of *P. aeruginosa* by alveolar macrophages. Our aforementioned results are similar to those reported by Shvedova et al. [12] following a single administration of 40 μg of SWCNTs in mice. However, they reported diminished clearance of *L. monocytogenes* following CNT exposure. In contrast, clearance of *P. aeruginosa* was not affected in our study. The lack of inhibition on pathogen clearance by nanoparticles, as shown in the current study, is not unique. For example, intratracheal inoculation of titanium dioxide nanorods in rats did not affect clearance of *L. monocytogenes* infection [28]. In fact, the clearance of *L.monocytogenes* was enhanced in rats exposed to silica [29]. Similarly, SWCNT exposure had no effect on the early immune response to *T. gondii* infection [13]. These results and ours demonstrate that the impact of airborne particulates on pulmonary immunity is not uniform and, in part, depends the type of particulate and the host-pathogen relationship.

Most research regarding CNT toxicity has focused on SWCNTs and MWCNTs while other CNT configurations including HCNTs and carbon nanohorns have received scant attention. Rapid adaptation and application of SWCNTs and MWCNTs into commercial products along with the development of bulk manufacturing plants to meet demand most likely accounts for this as it quickly raised awareness of these products in the toxicology community. Although the straight shapes of SWCNTs and MWCNTs allow for multiple applications, non-linear carbon nanotube configurations may be superior to straight nanotubes for specific roles. HCNTs possess similar mechanical properties as other CNTs such as semiconduction and high tensile strength. In addition, they show semimetallic characteristics not seen in straight CNTs [30] and it is hypothesized that HCNTs may also have superconductive properties. HCNTs have demonstrated an elastic spring-like behavior under tension [31] and could be developed as mechanical nanosprings. Additionally, the helical shape can enhance bonding strength between HCNTs greater than straight nanotubes, thus providing increased tensile strength [32]. HCNTs have been shown to be superior to SWCNTs as mechanical resonance sensors [33]. Conducting electricity through an HCNT results in the generation of an inductive magnetic field, which cannot be achieved in straight nanotubes, and thus HCNTs may be used to manufacture electromagnetic nanoswitches [32]. Understanding the potential toxicity of HCNTs would allow for the implementation of proper safety protocols prior to mass production.

The proinflammatory response and pulmonary lesions observed in our studies using HCNTs are quite similar to what has been reported in the literature in mice exposed to MWCNTs. In one study by Ronzani et al. a single administration of 25μg of MWCNTs resulted in elevated neutrophils and KC in the BAL exposed mice compared to controls similar to what we observed with our HCNTs. Repeat administrations (once every 7 days) of 25μg of MWCNTs over 21 days also resulted in elevated KC as well as macrophages, neutrophils, and lymphocytes in the BAL and histologic evaluation of MWCNT exposed lungs revealed granulomas and a minimal increase in collagen deposition around bronchioles [26]. Kim et al. also observed elevated neutrophils in the BAL of mice 24 hours after a single dose of 10μg or 100μg MWCNT as well as mildly elevated total cell counts in the BAL 2 weeks after administration and granulomatous inflammation in the 2 week post-exposure group [34]. Additional studies have documented the formation of pulmonary granulomas following MWCNT exposure [35,36]. Porter et al. reported elevations of neutrophils in the BAL of mice out to 56 days post MWCNT exposure [25]. Interestingly, exposure to MWCNTs resulted in the elevation of both total protein and LDH in the BAL whereas ours did not. A direct comparison of multiple studies may be difficult because of variables inherent to the specific MWCNTs used including diameter, length, purity, and production method. However, the host reaction to our HCNTs is similar to that of the straight MWCNTs with the exception that MWCNTs may be more cytotoxic than HCNTs as indicated by LDH and protein levels in the BAL.

Interpreting the impact of CNT exposure on clearance of pulmonary infections must take into account the pathogen used in the individual study. An effective immune response to *L. monocytogenes* infection requires phagocytosis, processing, and expression of antigens by macrophages to CD4 and CD8 T cells [37]. Thus interference with these functions by CNTs may significantly inhibit clearance. Neutrophils, however, are essential in the immune response of *P. aeruginosa* pulmonary infection and failure to do so results in elevated mortality [38,39]. During *early P. aeruginosa* infection, macrophages are essential for the release of neutrophil chemotactic factors [40,41] but the relative contribution of macrophage phagocytosis in clearance, although suggested to be important, remains to be elucidated in vivo. Our results demonstrate that CNT-induced elevation of both neutrophils and infiltrating macrophages (Figure 5C) may compensate for reduced macrophage phagocytosis, allowing for efficient clearance of extracellular pathogens. 

Interestingly, the lungs of mice preexposed to HCNTs and depleted of neutrophils contained significantly fewer PAO1 (1.5 log) compared to control-neutrophil depleted mice, contrary to what would be expected if HCNTs inhibited macrophage function. Examination of the BAL showed that the HCNT exposed mice contained more than twice as many macrophages than the control mice, suggesting that HCNT exposure induces macrophage chemotaxis following infection and the increased numbers of macrophages inhibit PAO1 proliferation. By depleting systemic macrophages with clodronate, control mice were better able to clear PAO1 infection than HCNT exposed mice, indicating that deficiencies in HCNT-laden alveolar macrophage function are also compensated by recruitment of systemic macrophages during infection.

Our exposure protocol is similar to those used in experiments demonstrating exacerbation of the immune system in allergy models by CNTs [6] as well the study involving the immune response to *T. gondii* [13]. A previous study has shown that approximately 50% of intranasally administered materials reach the lungs of mice [42]. Based on this assessment, we estimated the total HCNT deposition in the lungs to be ~25 µ per treatment and ~150 µg over the three weeks. Using previously published estimates of human minute ventilation during light work (20 L/minute) and a 40 hour work-week [43], deposition fraction of nano-sized particulates (30%) [25], mouse alveolar surface area (0.5 m^2^) and human alveolar surface area (102 m^2^) [25,44] calculated equivalent human deposition would be obtained in 8.4 years, 1.1 years, and 2.7 months when exposed to 53, 400, and 2000 μg/m^3^, respectively, representing airborne concentrations measured from facilities with various controls to limit airborne exposure [25,45,46]. Currently the Center for Disease Control/National Institute of Occupational Health and Safety recommends an exposure limit of 1 μg/m^3^ (NIOSH Current Intelligence Bulletin 65). At such a low concentration calculated equivalent human exposure would take ~ 443 years. However, as indicated above, facilities currently do not have control measures in place to achieve such a low exposure limit. Subchronic inhalation studies with rats showed a no-observed adverse effect level of 400 μg/m^3^ and, after adjusting for differences between rat and human lung anatomy and physiology to calculate human deposition equivalent, suggested an occupation exposure limit of 50 μg/m^3^ [47].

 One shortcoming of this estimation is accounting for clearance of HCNTs over time. One study in a rat model demonstrated that less than 0.15% of a single dose of 100 μg SWCNTs will translocate across the pulmonary epithelium [48]. A second rat study showed no change in the % of alveolar macrophages containing CNTs until 3 months after receiving one dose and can still found in macrophages after 6 months [49]. The presence of HCNTs was evident in more than 88% of alveolar macrophages in our study following 3 weeks of treatment (see Figure S8). Inhalation studies of carbon nanotubes have reported a biological half-life in the lungs from 51 days to 375 days depending on the airborn concentration and days of exposure has been reported [47,50,51]. Although it is unlikely that all 150 µg of HCNTs were present in the lungs at the end of our study, published studies and our observations evidence suggests that the majority of the nanotubes were still in the lungs. If we consider that less than 100% of the 150 µg dose is present, this would imply that the inflammatory response and alterations in phagocytosis resulted from a cumulative burden of less than 150 µg, suggesting that the equivalent human deposition would be obtained in less than 8.4 years, 1.1 years, or 2.7 months using the recorded airborne concentrations listed above. 

The elevated inflammatory response to pathogens and subsequent clearance may depend on the size of CNTs. Although no infection models comparing different CNTs have been published, results from ovalbumin sensitized mice suggest that longer MWCNTs are more proinflammatory than shorter CNTs [52,53]. Our HCNTs are similar in length to long MWCNTs and the elevated inflammatory response they induced, of which neutrophils predominate, may contribute to the clearance of extracellular Gram-negative organisms, compensating decreased phagocytosis by alveolar macrophages. In spite of the potential benefit of pathogen clearance, the elevated inflammatory response may also be detrimental to the health of the host secondary to pulmonary injury resulting from the enhanced neutrophil influx.

The mechanisms of CNT-mediated inhibition of phagocytosis remain elusive. Physical structure and dose of the CNTs used have been suggested to play a role. *In vitro* assays have reported that SWCNTs inhibit phagocytosis greater than MWCNTs at low concentrations [54] and that inhibition of phagocytosis by MWCNTs is both diameter and concentration dependent [55]. Shvedova et al. showed a concentration dependent inhibition of the phagocytosis of *L.monocytogenes* by alveolar macrophages *in vitro* [12]. However, in *vivo* phagocytosis was inhibited to a similar extent in both the low and high dose groups, suggesting that inhibition of phagocytosis and clearance of pulmonary pathogens by CNTs may involve additional mechanisms. 

## Conclusions

We have demonstrated that repeated exposure to HCNTs cause pulmonary granulomas, fibrosis, goblet cell hyperplasia, and elevates proinflammatory cytokines both pre- and post-infection. In addition, HCNTs inhibit phagocytosis of *P. aeruginosa* by alveolar macrophages.. However, a robust early immune response to *P. aeruginosa* observed in HCNT exposed mice compensated for this defect. This immune modulation appears to require the presence of macrophages, as control mice depleted of circulating macrophages were better able to clear infection than HCNT exposed mice. This model represents acute infection of mice exposed to HCNTs. Our results do not preclude the possibility that HCNT exposure may exacerbate chronic infection by *P. aeruginosa*. Our results and those of others show that the pulmonary response and clearance of pathogens following exposure to particulates is not uniform and differs by particle type and pathogen used. Thus, identifying and establishing relative risk will require studying the interactions between additional CNTs and pulmonary pathogens. 

## Supporting Information

Figure S1 **A representative SEM of dispersed nanotubes.** SEM shows moderate variability in the diameter of individual tubes as well as sharp kinks and folding.
(TIF)

Figure S2 **Determination of HCNT diameter by SEM analysis.** (**A- i to iv**) Typical SEM images of helical HCNT powder adhered on double sided carbon tape, and (**A- v and vi**) on a SiO2/Si substrate following vacuum filtration and transfer from a nitrocellulose membrane. (**B**) Histogram of HCNT diameter distribution obtained from the SEM images of A, showing an average diameter of ~200 nm with distribution (dHCNT = 199.9±127.8 nm, n=80) as indicated.
(TIF)

Figure S3 **Determination of HCNT length by SEM analysis.** (**A- i to vi**). Typical SEM images used to measure lengths of HCNTs. (**B**) Histogram of HCNT length distribution obtained from the SEM images of A, showing an average length of ~1.9 μm with distribution (LHCNT = 1.9±0.8 μm, n=50) as indicated.
(TIF)

Figure S4 **EDX analysis of the HCNTs on a Si surface.** The EDX reveals the Si substrate, C from the helical HCNTs, and trace elements of O and Fe from the transfer and growth processes, respectively. Inset: a SEM micrograph of the region under assessment by EDX. HCNTs and tape residues are apparent.
(TIF)

Figure S5 **XPS analysis of HCNTs.** (**A**) XPS survey of HCNT Powder. (**B**) High resolution C 1s XPS spectra shows components at 284.5 eV (Helical CNTs) and ~290 (satellite peak for graphitic carbon). Scatter points are raw data and solid lines are fits and background. (**C**) High resolution Cl 2p XPS spectra. (**D**) High resolution Al 2p XPS spectra.
(TIF)

Figure S6 **Raman spectroscopic analysis of HCNTs.** Raman spectroscopy data for helical HCNT powder showing peaks related to the doubly degenerate optical phonon mode at the Brillouin zone center (G-peak) in graphitic materials, and a disorder-induced peak related to defects in the crystal structure (D-peak)(8).
(TIF)

Figure S7 **DLS analysis of HCNTs.** Dynamic light scattering of HCNTs dispersed in our media show a mean diameter of 532 nm.
(TIF)

Figure S8 **Alveolar macrophage phagocytosis of HCNTs.** Evaluation of macrophages from the BAL of mice exposed to HCNTs shows an average of 71.5% of macrophages containing HCNTs in their cytoplasm following a single exposure while 88% of macrophages have phagocytized HCNTs after 3 weeks of repeated exposure.
(TIF)

Text S1 Supporting text.
(PDF)

## References

[1] Saito R, Dresselhaus G, Dresselhaus M (1998) Physical properties of carbon nanotubes. London: Imperial College Press pp. 1-70.

[2] Lam Chiu-Wing, James JT, McCluskey R, Arepalli S, Hunter RL (2006) A review of carbon nanotube toxicity and assessment of potential occupational and environmental health risks. Crit Rev Toxicol 36: 189-217. doi:10.1080/10408440600570233. PubMed: 16686422. 16686422

[3] Shvedova AA, Kisin ER, Porter D, Schulte P, Kagan VE et al. (2009) Mechanisms of pulmonary toxicity and medical applications of carbon nanotubes: Two faces of janus? Pharmacol Ther 121: 192-204. doi:10.1016/j.pharmthera.2008.10.009. PubMed: 19103221. 19103221

[4] Pacurari M, Qian Y, Porter DW, Wolfarth M, Wan Y et al. (2011) Multi-walled carbon nanotube-induced gene expression in the mouse lung: Association with lung pathology. Toxicol Appl Pharmacol 255: 18-31. doi:10.1016/j.taap.2011.05.012. PubMed: 21624382. 21624382 PMC3148292

[5] Wang P, Nie X, Wang Y, Li Y, Ge C et al. (2013) Multiwall carbon nanotubes mediate macrophage activation and promote pulmonary fibrosis through TGF-β/Smad signaling pathway. Small: ([MedlinePgn:]) doi:10.1002/smll.201300607. [Epub ahead of print] PubMed: 23650105. 23650105

[6] Inoue K, Yanagisawa R, Koike E, Nishikawa M, Takano H (2010) Repeated pulmonary exposure to single-walled carbon nanotubes exacerbates allergic inflammation of the airway: Possible role of oxidative stress. Free Radic Biol Med 48: 924-934. doi:10.1016/j.freeradbiomed.2010.01.013. PubMed: 20093178. 20093178

[7] Ryman-Rasmussen JP, Tewksbury EW, Moss OR, Cesta MF, Wong BA et al. (2009) Inhaled multiwalled carbon nanotubes potentiate airway fibrosis in murine allergic asthma. Am J Respir Cell Mol Biol 40: 349-358. doi:10.1165/rcmb.2008-0276OC. PubMed: 18787175. 18787175 PMC2645533

[8] Herzog E, Byrne HJ, Casey A, Davoren M, Lenz AG et al. (2009) SWCNT suppress inflammatory mediator responses in human lung epithelium in vitro. Toxicol Appl Pharmacol 234: 378-390. doi:10.1016/j.taap.2008.10.015. PubMed: 19041333. 19041333

[9] Mitchell LA, Gao J, Wal RV, Gigliotti A, Burchiel SW et al. (2007) Pulmonary and systemic immune response to inhaled multiwalled carbon nanotubes. Toxicol Sci 100: 203-214. doi:10.1093/toxsci/kfm196. PubMed: 17660506. 17660506

[10] Mitchell LA, Lauer FT, Burchiel SW, McDonald JD (2009) Mechanisms for how inhaled multiwalled carbon nanotubes suppress systemic immune function in mice. Nat Nanotechnol 4: 451-456. doi:10.1038/nnano.2009.151. PubMed: 19581899. 19581899 PMC3641180

[11] Tkach AV, Shurin GV, Shurin MR, Kisin ER, Murray AR et al. (2011) Direct effects of carbon nanotubes on dendritic cells induce immune suppression upon pulmonary exposure. ACS Nano 5: 5755-5762. doi:10.1021/nn2014479. PubMed: 21657201. 21657201 PMC3170729

[12] Shvedova AA, Fabisiak JP, Kisin ER, Murray AR, Roberts JR et al. (2008) Sequential exposure to carbon nanotubes and bacteria enhances pulmonary inflammation and infectivity. Am J Respir Cell Mol Biol 38: 579-590. doi:10.1165/rcmb.2007-0255OC. PubMed: 18096873. 18096873 PMC2335338

[13] Swedin L, Arrhigi R, Andersson-Willman B, Murray A, Chen Y et al. (2012) Pulmonary exposure to single-walled carbon nanotubes does not affect the early immune response against toxoplasma gondii. Part Fibres Toxicol 9: 16. doi:10.1186/1743-8977-9-16. PMC3495637 22621311

[14] Diekema DJ, Pfaller MA, Jones RN, Doern GV, Winokur PL et al. (1999) Survey of bloodstream infections due to gram-negative bacilli: Frequency of occurrence and antimicrobial susceptibility of isolates collected in the united states,canada, and latin america for the SENTRY antimicrobial surveillance program, 1997. Clin Infect Dis 29: 595-607. doi:10.1086/598640. PubMed: 10530454. 10530454

[15] Kuang Z, Hao Y, Walling BE, Jeffries JL, Ohman DE et al. (2011) *Pseudomonas* *aeruginosa* elastase provides an escape from phagocytosis by degrading the pulmonary surfactant protein-A. PLOS ONE 6: e27091. doi:10.1371/journal.pone.0027091. PubMed: 22069491. 22069491 PMC3206073

[16] Zhang S, McCormack FX, Levesque RC, O'Toole GA, Lau GW (2007) The flagellum of *pseudomonas* *aeruginosa* is required for resistance to clearance by surfactant protein A. PLOS ONE 2: e564. doi:10.1371/journal.pone.0000564. PubMed: 17593964. 17593964 PMC1891440

[17] Hao Y, Kuang Z, Walling BE, Bhatia S, Sivaguru M et al. (2012) Pseudomonas aeruginosa pyocyanin causes airway goblet cell hyperplasia and metaplasia and mucus hypersecretion by inactivating the transcriptional factor FoxA2. Cell Microbiol 14: 401-415. doi:10.1111/j.1462-5822.2011.01727.x. PubMed: 22103442. 22103442

[18] Meijering E, Jacob M, Sarria J-F, Steiner P, Hirling H et al. (2004) Design and validation of a tool for neurite tracing and analysis in fluorescence microscopy images. Cytometry A 58A: 167-176. doi:10.1002/cyto.a.20022. PubMed: 15057970. 15057970

[19] Peigney A, Laurent C, Flahaut E, Bacsa RR, Rousset A (2001) Specific surface area of carbon nanotubes and bundles of carbon nanotubes. Carbon 39: 507-514. doi:10.1016/S0008-6223(00)00155-X.

[20] Hu X, Cook S, Wang P, Hwang HM, Liu X et al. (2010) In vitro evaluation of cytotoxicity of engineered carbon nanotubes in selected human cell lines. Sci Total Environ 408: 1812-1817. doi:10.1016/j.scitotenv.2010.01.035. PubMed: 20167353. 20167353

[21] Mercer RR, Hubbs AF, Scabilloni JF, Wang L, Battelli LA et al. (2011) Pulmonary fibrotic response to aspiration of multi-walled carbon nanotubes. Part Fibres Toxicol 8: 21. doi:10.1186/1743-8977-8-21. PubMed: 21781304. PMC3152886 21781304

[22] Mercer RR, Scabilloni J, Wang L, Kisin E, Murray AR et al. (2008) Alteration of deposition pattern and pulmonary response as a result of improved dispersion of aspirated single-walled carbon nanotubes in a mouse model. Am J Physiol Lung Cell Mol Physiol 294: L87-L97. PubMed: 18024722. 18024722 10.1152/ajplung.00186.2007

[23] Shvedova AA, Kisin E, Murray AR, Johnson VJ, Gorelik O et al. (2008) Inhalation vs. aspiration of single-walled carbon nanotubes in C57BL/6 mice: Inflammation, fibrosis, oxidative stress, and mutagenesis. Am J Physiol Lung Cell Mol Physiol 295: L552-L565. doi:10.1152/ajplung.90287.2008. PubMed: 18658273. 18658273 PMC2575941

[24] Chou CC, Hsiao HY, Hong QS, Chen CH, Peng YW et al. (2008) Single-walled carbon nanotubes can induce pulmonary injury in mouse model. Nano Lett 8: 437-445. doi:10.1021/nl0723634. PubMed: 18225938. 18225938

[25] Porter DW, Hubbs AF, Mercer RR, Wu N, Wolfarth MG et al. (2010) Mouse pulmonary dose- and time course-responses induced by exposure to multi-walled carbon nanotubes. Toxicology 269: 136-147. doi:10.1016/j.tox.2009.10.017. PubMed: 19857541. 19857541

[26] Ronzani C, Spiegelhalter C, Vonesch JL, Lebeau L, Pons F (2012) Lung deposition and toxicological responses evoked by multi-walled carbon nanotubes dispersed in a synthetic lung surfactant in the mouse. Arch Toxicol 86: 137-149. doi:10.1007/s00204-011-0741-y. PubMed: 21805258. 21805258

[27] Inoue K, Koike E, Yanagisawa R, Hirano S, Nishikawa M et al. (2009) Effects of multi-walled carbon nanotubes on a murine allergic airway inflammation model. Toxicol Appl Pharmacol 237: 306-316. doi:10.1016/j.taap.2009.04.003. PubMed: 19371758. 19371758

[28] Roberts JR, Chapman RS, Tirumala VR, Karim A, Chen BT et al. (2011) Toxicological evaluation of lung responses after intratracheal exposure to non-dispersed titanium dioxide nanorods. J Toxicol Environ Health A 74: 790-810. doi:10.1080/15287394.2011.567954. PubMed: 21541881. 21541881

[29] Antonini JM, Roberts JR, Yang HM, Barger MW, Ramsey D et al. (2000) Effect of silica inhalation on the pulmonary clearance of a bacterial pathogen in fischer 344 rats. Lung 178: 341-350. doi:10.1007/s004080000038. PubMed: 11361057. 11361057

[30] Akagi K, Tamura R, Tsukada M, Itoh S, Ihara S (1995) Electronic structure of helically coiled cage of graphitic carbon. Phys Rev Lett 74: 2307-2310. doi:10.1103/PhysRevLett.74.2307. PubMed: 10057895. 10057895

[31] Chen X, Zhang S, Dikin DA, Ding W, Ruoff RS, Pan L, Nakayama Y (2003) Mechanics of a carbon nanocoil. Nano Lett 3: 1299-1304. doi:10.1021/nl034367o.

[32] Lau KT, Lu M, Hui D (2006) Coiled carbon nanotubes: Synthesis and their potential applications in advanced composite structures. Composites B: Eng 37: 437-448. doi:10.1016/j.compositesb.2006.02.008.

[33] Volodin A, Buntinx D, Ahlskog M, Fonseca A, Nagy JB et al. (2004) Coiled carbon nanotubes as self-sensing mechanical resonators. Nano Lett 4: 1775-1779. doi:10.1021/nl0491576.

[34] Kim JE, Lim HT, Minai-Tehrani A, Kwon JT, Shin JY et al. (2010) Toxicity and clearance of intratracheally administered multiwalled carbon nanotubes from murine lung. J Toxicol Environ Health A 73: 1530-1543. doi:10.1080/15287394.2010.511578. PubMed: 20954079. 20954079

[35] Li JG, Li WX, Xu JY, Cai XQ, Liu RL et al. (2007) Comparative study of pathological lesions induced by multiwalled carbon nanotubes in lungs of mice by intratracheal instillation and inhalation. Environ Toxicol 22: 415-421. doi:10.1002/tox.20270. PubMed: 17607736. 17607736

[36] Huizar I, Malur A, Midgette YA, Kukoly C, Chen P et al. (2011) Novel murine model of chronic granulomatous lung inflammation elicited by carbon nanotubes. Am J Respir Cell Mol Biol 45: 858-866. doi:10.1165/rcmb.2010-0401OC. PubMed: 21398620. 21398620 PMC5460893

[37] Shen H, Tato CM, Fan X (1998) Listeria monocytogenes as a probe to study cell-mediated immunity. Curr Opin Immunol 10: 450-458. doi:10.1016/S0952-7915(98)80120-9. PubMed: 9722922. 9722922

[38] Koh AY, Priebe GP, Ray C, Van Rooijen N, Pier GB (2009) Inescapable need for neutrophils as mediators of cellular innate immunity to acute pseudomonas aeruginosa pneumonia. Infect Immun 77: 5300-5310. doi:10.1128/IAI.00501-09. PubMed: 19805527. 19805527 PMC2786465

[39] Scarff JM, Goldberg JB (2008) Vaccination against *Pseudomonas* *aeruginosa* pneumonia in immunocompromised mice. Clin Vaccine Immunol 15: 367–375. doi:10.1128/CVI.00419-07. PubMed: 18094113. 18094113 PMC2238050

[40] Kooguchi K, Hashimoto S, Kobayashi A, Kitamura Y, Kudoh I et al. (1998) Role of alveolar macrophages in initiation and regulation of inflammation in *Pseudomonas* *aeruginosa* pneumonia. Infect Immun 66: 3164-3169. PubMed: 9632581. 9632581 10.1128/iai.66.7.3164-3169.1998 PMC108328

[41] Hashimoto S, Pittet JF, Hong K, Folkesson H, Bagby G et al. (1996) Depletion of alveolar macrophages decreases neutrophil chemotaxis to Pseudomonas airspace infections. Am J Physiol 270: L819-L828. PubMed: 8967517. 8967517 10.1152/ajplung.1996.270.5.L819

[42] Southam DS, Dolovich M, O'Byrne PM, Inman MD (2002) Distribution of intranasal instillations in mice: Effects of volume, time, body position, and anesthesia. Am J Physiol Lung Cell Mol Physiol 282: L833-L839. PubMed: 11880310. 11880310 10.1152/ajplung.00173.2001

[43] Galer DM, Leung HW, Sussman RG, Trzos RJ (1992) Scientific and practical considerations for the development of occupational exposure limits (OELs) for chemical substances. Regul Toxicol Pharmacol 15: 291-306. doi:10.1016/0273-2300(92)90040-G. PubMed: 1509122. 1509122

[44] Stone KC, Mercer RR, Gehr P, Stockstill B, Crapo JD (1992) Allometric relationships of cell numbers and size in the mammalian lung. Am J Respir Cell Mol Biol 6: 235-243. doi:10.1165/ajrcmb/6.2.235. PubMed: 1540387. 1540387

[45] Kuempel ED (2011) Carbon nanotube risk assessment: implications for exposure and medical monitoring. J Occup Environ Med 53: S91-S97. doi:10.1097/JOM.0b013e31821b1f3f. PubMed: 21654426. 21654426

[46] Han JH, Lee EJ, Lee JH, So KP, Lee YH et al. (2008) Monitoring multiwalled carbon nanotube exposure in carbon nanotube research facility. Inhal Toxicol 20: 741-749. doi:10.1080/08958370801942238. PubMed: 18569096. 18569096

[47] Pauluhn J (2010) Multi-walled carbon nanotubes (baytubes): Approach for derivation of occupational exposure limit. Regul Toxicol Pharmacol 57: 78-89. doi:10.1016/j.yrtph.2009.12.012. PubMed: 20074606. 20074606

[48] Matthews IP, Gregory CJ, Aljayyoussi G, Morris CJ, McDonald I et al. (2013) Maximal extent of translocation of single-walled carbon nanotubes from lung airways of the rat. Environ Toxicol Pharmacol 35: 461-464. doi:10.1016/j.etap.2013.02.002. PubMed: 23501606. 23501606

[49] Elgrabli D, Floriani M, Abella-Gallart S, Meunier L, Gamez C et al. (2008) Biodistribution and clearance of instilled carbon nanotubes in rat lung. Part Fibres Toxicol 5: 20-20. doi:10.1186/1743-8977-5-20. PubMed: 19068117. PMC2645433 19068117

[50] Ellinger-Ziegelbauer H, Pauluhn J (2009) Pulmonary toxicity of multi-walled carbon nanotubes (baytubes) relative to alpha-quartz following a single 6h inhalation exposure of rats and a 3 months post-exposure period. Toxicology 266: 16-29. doi:10.1016/j.tox.2009.10.007. PubMed: 19836432. 19836432

[51] Oyabu T, Myojo T, Morimoto Y, Ogami A, Hirohashi M et al. (2011) Biopersistence of inhaled MWCNT in rat lungs in a 4-week well-characterized exposure. Inhal Toxicol 23: 784-791. doi:10.3109/08958378.2011.608096. PubMed: 22035120. 22035120

[52] Erdely A, Hulderman T, Salmen R, Liston A, Zeidler-Erdely PC et al. (2009) Cross-talk between lung and systemic circulation during carbon nanotube respiratory exposure. potential biomarkers. Nano Lett 9: 36-43. doi:10.1021/nl801828z. PubMed: 19049393. 19049393

[53] Nygaard UC, Hansen JS, Samuelsen M, Alberg T, Marioara CD et al. (2009) Single-walled and multi-walled carbon nanotubes promote allergic immune responses in mice. Toxicol Sci 109: 113-123. doi:10.1093/toxsci/kfp057. PubMed: 19293371. 19293371

[54] Jia G, Wang H, Yan L, Wang X, Pei R et al. (2005) Cytotoxicity of carbon nanomaterials: Single-wall nanotube, multi-wall nanotube, and fullerene. Environ Sci Technol 39: 1378-1383. doi:10.1021/es048729l. PubMed: 15787380. 15787380

[55] Wang X, Jia G, Wang H, Nie H, Yan L et al. (2009) Diameter effects on cytotoxicity of multi-walled carbon nanotubes. J Nanosci Nanotechnol 9: 3025-3033. doi:10.1166/jnn.2009.025. PubMed: 19452965. 19452965

