# Supplementary figures and images for: Helical Carbon Nanotubes Enhance the Early Immune Response and Inhibit Macrophage-Mediated Phagocytosis of Pseudomonas aeruginosa
Source: PLoS One. 2013 Nov 18;8(11):e80283. doi: 10.1371/journal.pone.0080283 (PMC3855819; doi:10.1371/journal.pone.0080283)

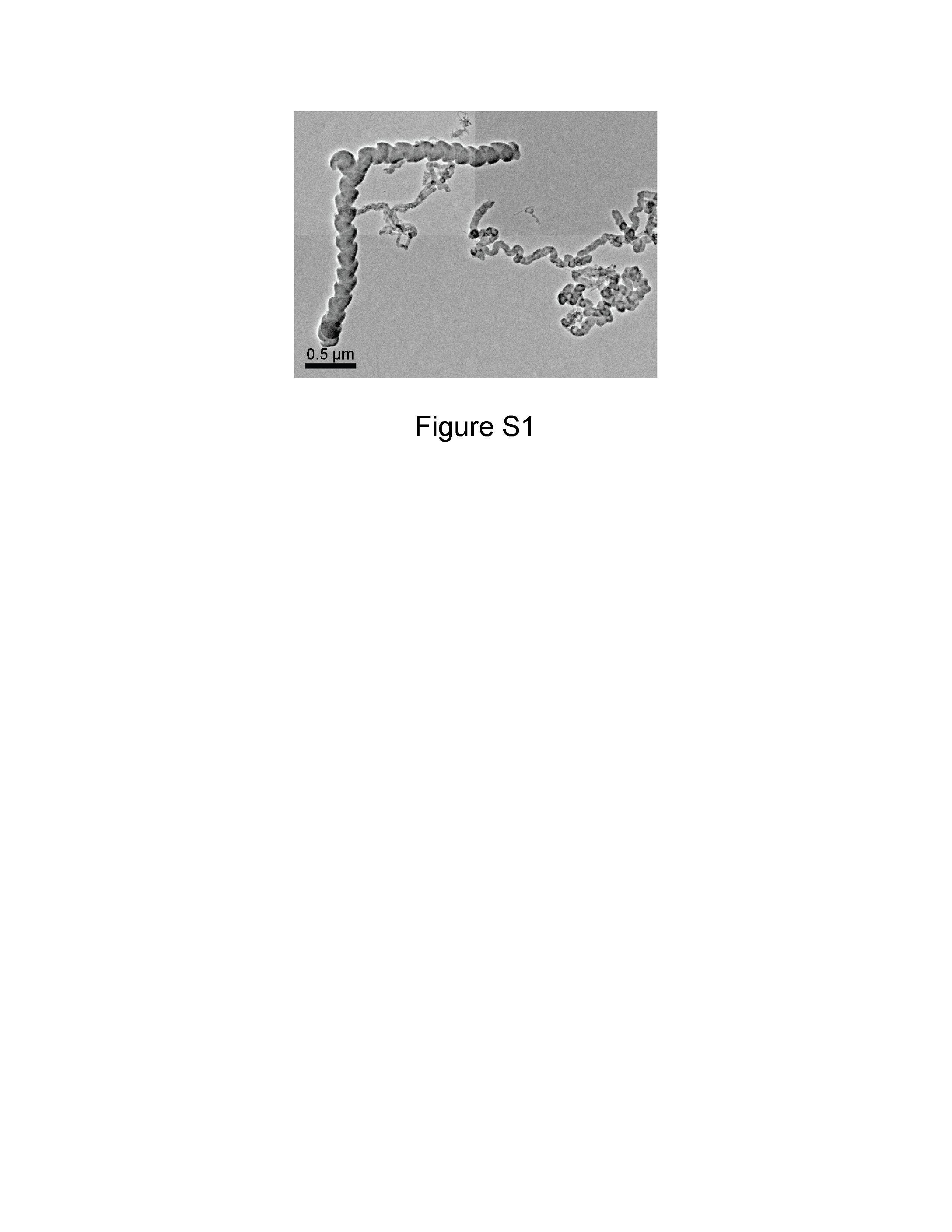

Supplement: Figure S1 — A representative SEM of dispersed nanotubes. SEM shows moderate variability in the diameter of individual tubes as well as sharp kinks and folding. (TIF) [file pone.0080283.s001.tif]

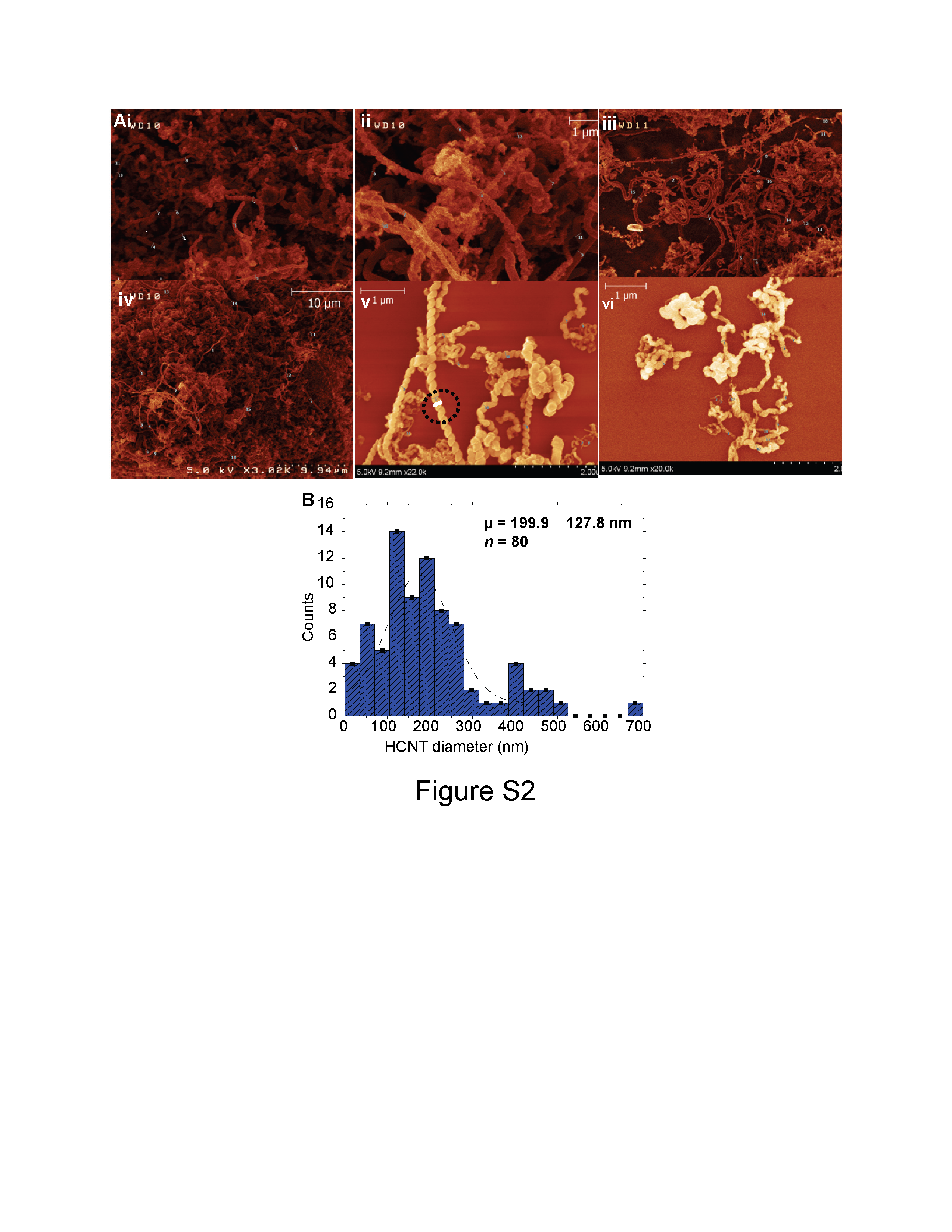

Supplement: Figure S2 — Determination of HCNT diameter by SEM analysis. (A- i to iv) Typical SEM images of helical HCNT powder adhered on double sided carbon tape, and (A- v and vi) on a SiO2/Si substrate following vacuum filtration and transfer from a nitrocellulose membrane. (B) Histogram of HCNT diameter distribution obtained from the SEM images of A, showing an average diameter of ~200 nm with distribution (dHCNT = 199.9±127.8 nm, n=80) as indicated. (TIF) [file pone.0080283.s002.tif]

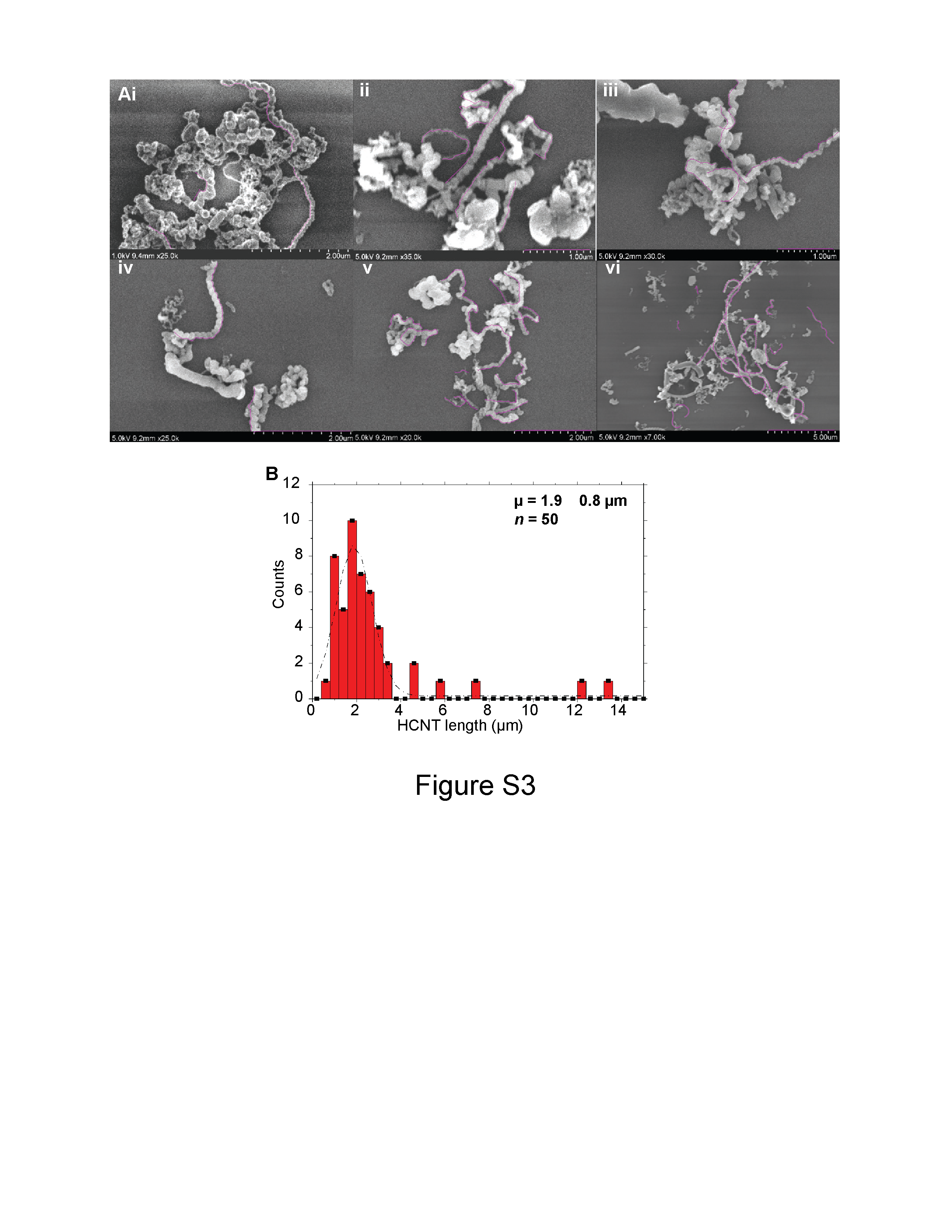

Supplement: Figure S3 — Determination of HCNT length by SEM analysis. (A- i to vi). Typical SEM images used to measure lengths of HCNTs. (B) Histogram of HCNT length distribution obtained from the SEM images of A, showing an average length of ~1.9 μm with distribution (LHCNT = 1.9±0.8 μm, n=50) as indicated. (TIF) [file pone.0080283.s003.tif]

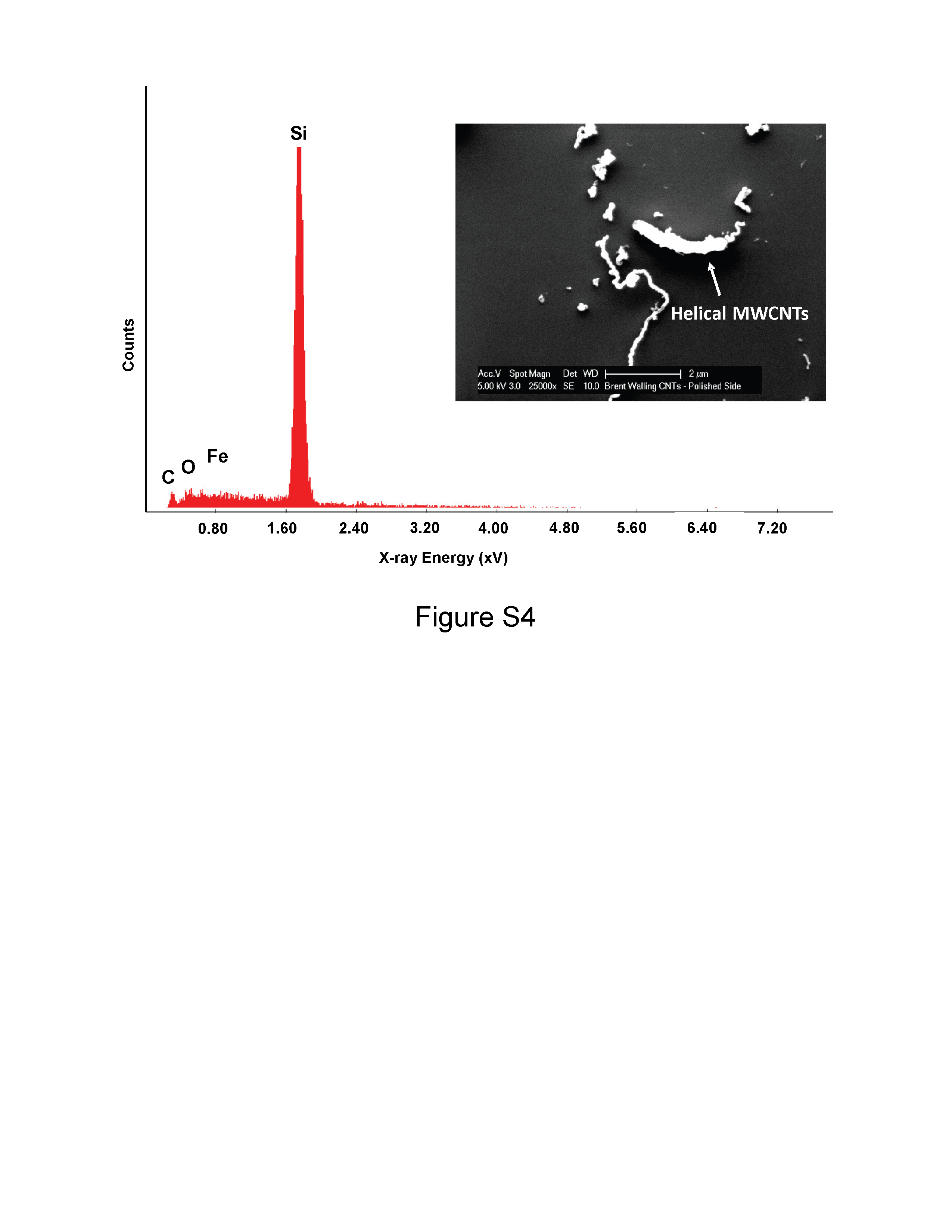

Supplement: Figure S4 — EDX analysis of the HCNTs on a Si surface. The EDX reveals the Si substrate, C from the helical HCNTs, and trace elements of O and Fe from the transfer and growth processes, respectively. Inset: a SEM micrograph of the region under assessment by EDX. HCNTs and tape residues are apparent. (TIF) [file pone.0080283.s004.tif]

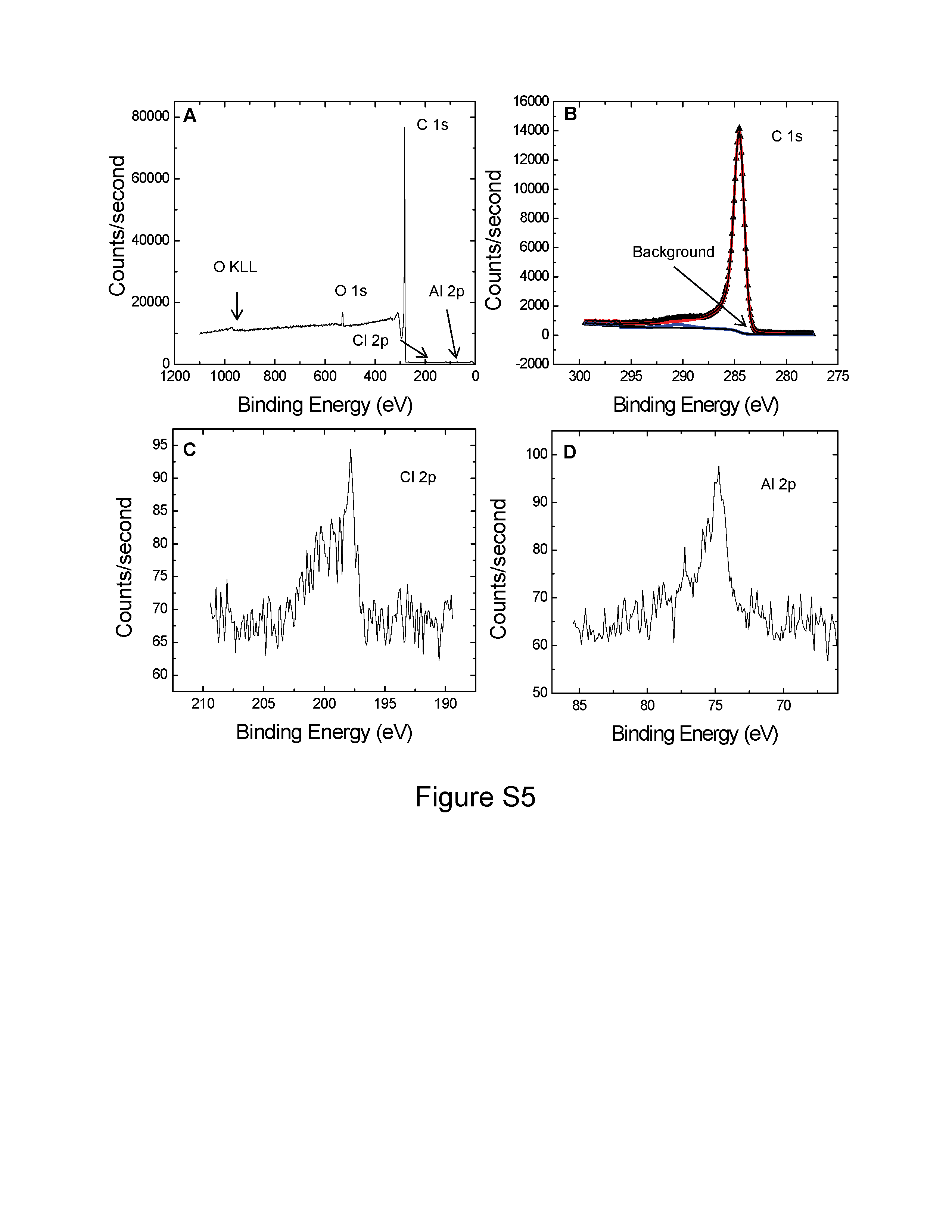

Supplement: Figure S5 — XPS analysis of HCNTs. (A) XPS survey of HCNT Powder. (B) High resolution C 1s XPS spectra shows components at 284.5 eV (Helical CNTs) and ~290 (satellite peak for graphitic carbon). Scatter points are raw data and solid lines are fits and background. (C) High resolution Cl 2p XPS spectra. (D) High resolution Al 2p XPS spectra. (TIF) [file pone.0080283.s005.tif]

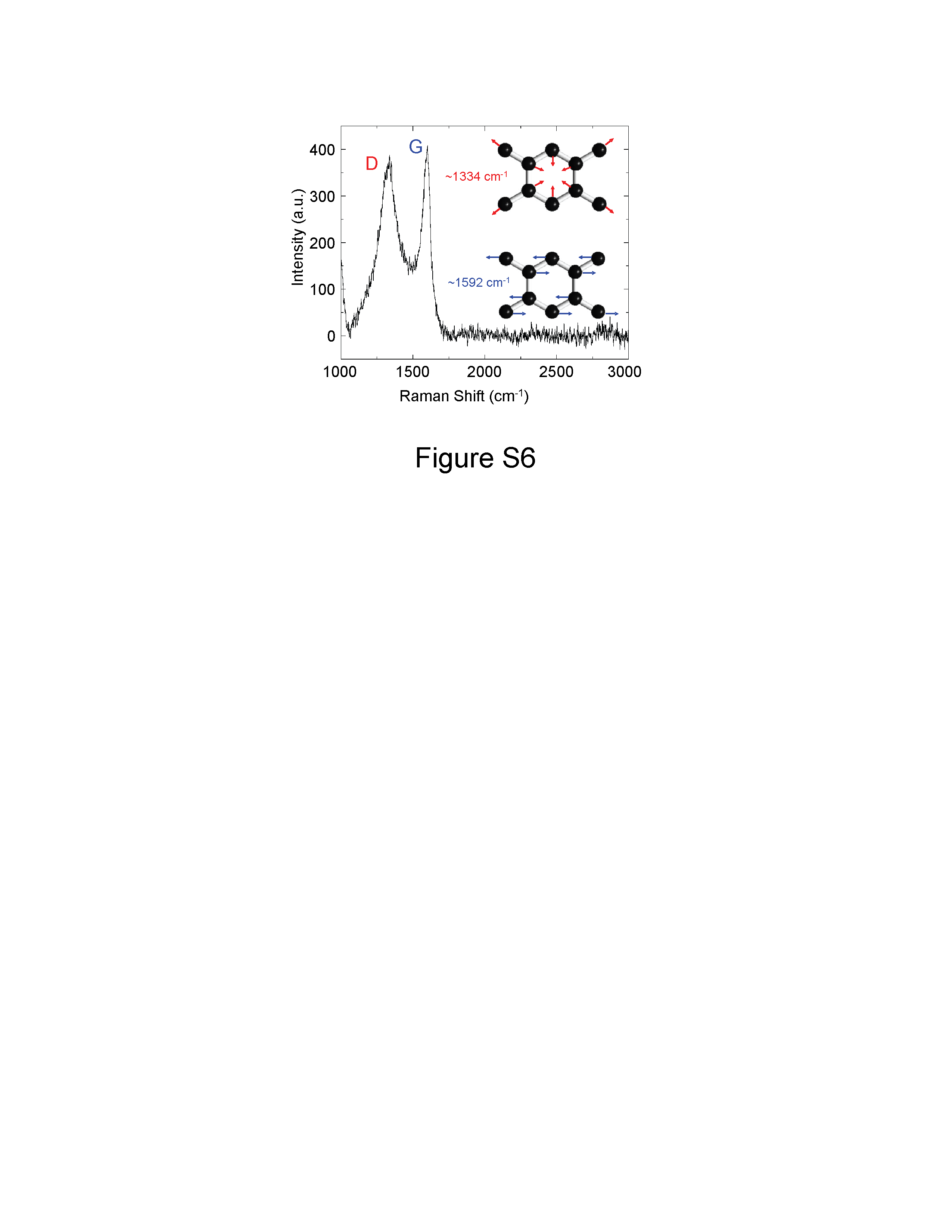

Supplement: Figure S6 — Raman spectroscopic analysis of HCNTs. Raman spectroscopy data for helical HCNT powder showing peaks related to the doubly degenerate optical phonon mode at the Brillouin zone center (G-peak) in graphitic materials, and a disorder-induced peak related to defects in the crystal structure (D-peak)(8). (TIF) [file pone.0080283.s006.tif]

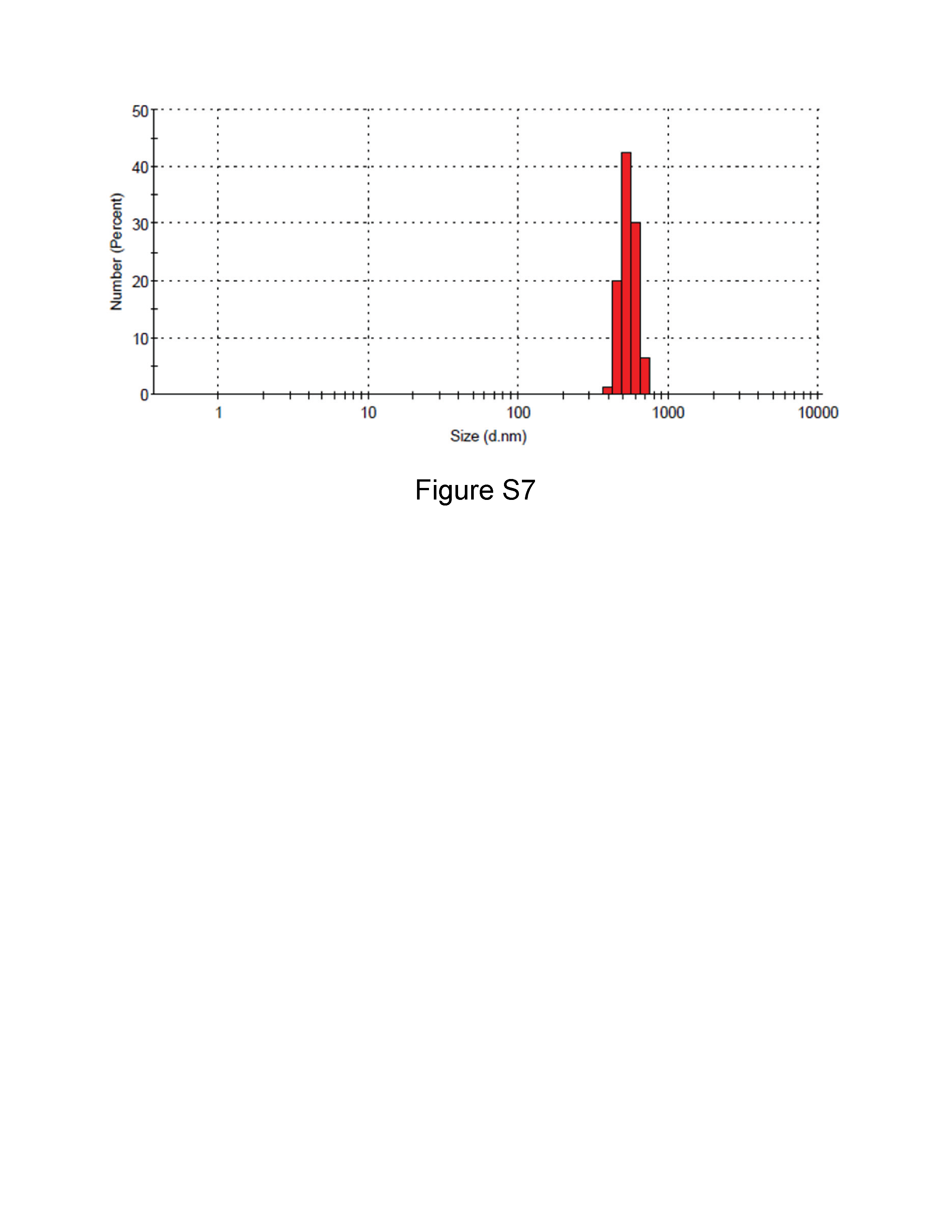

Supplement: Figure S7 — DLS analysis of HCNTs. Dynamic light scattering of HCNTs dispersed in our media show a mean diameter of 532 nm. (TIF) [file pone.0080283.s007.tif]

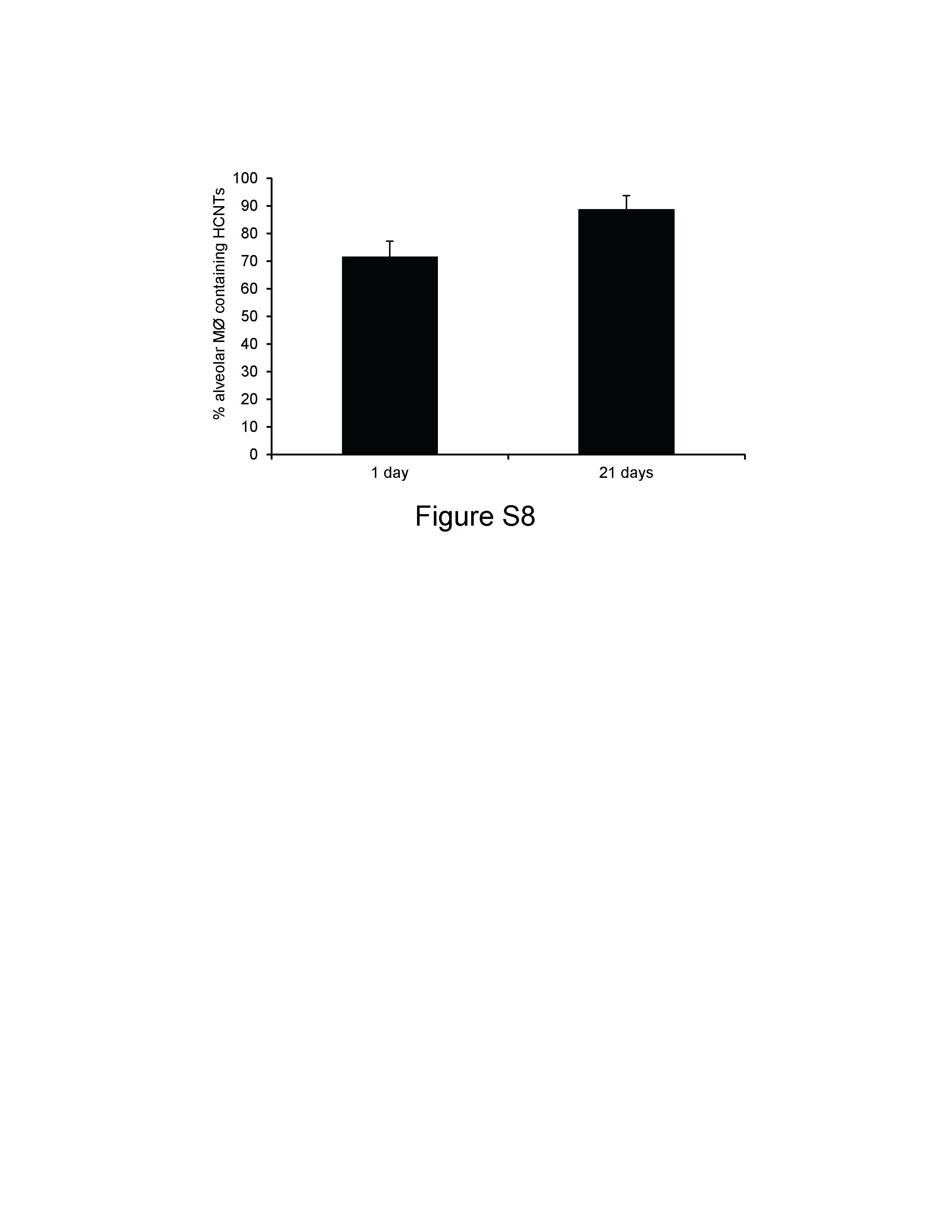

Supplement: Figure S8 — Alveolar macrophage phagocytosis of HCNTs. Evaluation of macrophages from the BAL of mice exposed to HCNTs shows an average of 71.5% of macrophages containing HCNTs in their cytoplasm following a single exposure while 88% of macrophages have phagocytized HCNTs after 3 weeks of repeated exposure. (TIF) [file pone.0080283.s008.tif]
