# Supplementary material for: Helical Carbon Nanotubes Enhance the Early Immune Response and Inhibit Macrophage-Mediated Phagocytosis of Pseudomonas aeruginosa
Source: PLoS One. 2013 Nov 18;8(11):e80283. doi: 10.1371/journal.pone.0080283 (PMC3855819; doi:10.1371/journal.pone.0080283)
Supplement: Text S1 — Supporting text. (PDF) [file pone.0080283.s009.pdf]

Supplementary Online Material

Helical Carbon Nanotubes Inhibit Macrophage-Mediated Phagocytosis of *Pseudomonas aeruginosa*

MATERIALS AND METHODS

Chemicals and cell lines

All chemicals were purchased from Sigma-Aldrich (St. Louis, MO), unless stated otherwise.

Preparation of helical carbon nanotubes (HCNTs)

HCNTs were obtained from Cheap Tubes Inc. (Brattleboro,Vermont). For all studies, dry HCNTs were suspended to 1 mg/ml in DMEM without phenol (Gibco, Grand Island, NY) with PBS with 0.01 % Tween-80. Immediately prior to use, nanotubes were vortexed 30 seconds, sonicated with a Soniprep 150 (23 KHz frequency,6 micron amplitude) on ice for 5 minutes with a 30 second on-30 second off cycle, and diluted to desired concentrations. All nanotubes were used within 30 minutes of sonication and vortexed just prior to use. The enotoxin concentration was determined by the Limulus amebocyte assay (ToxinSensor Chromogenic LAL Endotoxin Assay Kit, GenScript, Piscataway, NJ, detection limit 0.005 EU/ml). Endotoxin levels of HCNTs in dispersal media were < 0.005 EU/ml. Dispersion following sonication was determined by measuring light absorption at 550 nm wavelength.

HCNT characterization

Bright field transmission electron microscopy (TEM) images were acquired on a JEOL JEM2100 microscope operated at 200 kV with a spherical aberration coefficient of 2.0 mm (Fig. S1). The nanotubes were prepared by sonicating in ethyl alcohol for 5 minutes on ice, vortexed, and dispersed on copper grids coated with Formvar plastic. A Hitachi S-4800 field emission scanning electron microscope (SEM) was used to analyze the structural features of HCNTs. HCNTs were pressed onto double-sided carbon tape and imaged under a 5.0 kV accelerating voltage at varying magnifications (Fig. S2-A i-iv). HCNTs were also dispersed in 2% wt/vol sodium dodecyl sulfate (SDS) by centrifugation followed by vacuum filtration onto nitrocellulose membranes. The HCNTs were then transferred to a SiO_2/Si supporting substrate for SEM analysis (Fig. S2-A v and vi). The diameter distribution was then obtained by importing the SEM files into Gwyddion (http://gwyddion.net/) [1]. Line profiles were taken perpendicular to the longitudinal axis of the HCNTs, ensuring that the profile ended at the HCNT edge. An example line profile is encircled in Fig. S2-A v. The HCNTs have a diameter distribution of 50-500 nm, normally distributed about ~200 nm (n = 80) (Fig. S2-B).

Additionally, we determine the lengths of the helical HCNTs by edge extraction with NeuronJ [2]. We use SEM images gathered in the same way as Fig. S1A i-vi to extract the HCNT length. By analyzing the distance between maxima in the HCNTs' helices, we calculate the helical pitch to be b ≈ 12 nm. With the SEM images in Fig. S3A i-vi and NeuronJ, the apparent HCNT length is L_{app} = 1.40 ± 0.53 µm (n = 50). To determine the actual HCNT length, that is, the length of the unraveled helix, we employ

the expression $L_{\mathrm{HCNT}} = \left[L_{\mathrm{app}} / \; 2\pi b \; \right] \sqrt{a^2 + b^2}$, where *a* is the HCNT radius (~100 nm) and *b* is the helical pitch (~12 nm). Therefore, the helical length distribution is L_{HCNT} = 1.9 ± 0.8 µm, as shown in Fig. S3-B. The difference between the helical length L_{HCNT} and the apparent length L_{app} is $\Delta L = L_{\mathrm{HCNT}} - L_{\mathrm{app}}$. ΔL ranges from 0 to 1.46 µm, with a mean at 0.5 µm.

Using the diameter and length information, we can estimate the specific surface area (SSA) of the HCNTs. The interior core of HCNTs has previously been shown to be hollow [3]. There is a dense array of multi-walled CNTs (MWNTs) that surrounds this hollow core, and the hollow core is ~2 nm in dimension [3,4]. The thickness of a MWCNT is that of graphene (0.34 nm) and the separation between walls in a MWNT is ~0.35 nm [4](Saito et al., 1998). Therefore we can calculate the number of shells within the HCNTs by subtracting the hollow core's contribution from the radius distribution (R_{HCNT} = 100.0 ± 63.9 nm) and dividing by 0.7 nm per shell. We estimate the number of shells to be N_{S} = 143 +/- 91. The specific surface area (SSA), is then given by the modified [5] equation:

$$SSA = \frac{d_{HCNT} \;\; 1315 + \pi \Delta L}{d_{HCNT} N_S - 0.68 \sum_{i}^{N_S - 1} i} \quad \text{units: } \frac{\mathrm{m}^2}{\mathrm{g}} . \tag{1}$$

From eq. (1), the number of shells N_{s}, the diameter distribution d_{HCNT}, and the length distribution ΔL, the range of SSA values is *SSA* = 38.6 ± 28.7 m^2/g. Comparatively, the *bulk* SSA value from the manufacturer, extracted by Brunauer-Emmett-Teller (BET) analysis, was *SSA* >30 m^2/g (www.cheaptubesinc.com).

Elemental analysis of small regions of the HCNTs was performed by energy dispersive X-ray spectroscopy (EDX) using a Philips XL30 environmental SEM (ESEM)

with an EDX column. Samples were prepared by impregnating a Si surface with a piece of Scotch tape covered with HCNT powder. EDX (Fig. S4) reveals the Si substrate and the presence of C. The O and Fe signals are weak and just above the noise floor of the instrument. Still, we hold that these are real, and thus there are trace amounts of O and Fe within the HCNTs. The O likely resulted from the oxidized Si surface and adsorbed O_2 on the HCNTs. It is also possible that the HCNTs' sidewall defects could be terminated with oxygen. The Fe results from the growth processes involved in generating the HWCNTs.

Additional chemical analysis of HCNTs was performed by X-ray photoelectron spectroscopy (XPS) using a KRATOS Axis Ultra 165 mm X-ray photoelectron spectrometer, and carried out on a small amount of HCNTs deposited on vacuum compatible 3M copper conductive tape (Pella product no. 16074). The XPS survey spectrum (Fig. S5-A) shows the presence of O, C, Cl, and Al. The presence of O is likely due to adsorbed water, as the XPS survey does not indicate the presence of carbon functional groups related to the poly-acrylate adhesive of the conductive copper tape used during XPS analysis. High resolution C-1s XPS spectra (Fig. S5-B) shows components at 284.5 eV (HCNTs) and 290 eV (satellite peak for graphitic carbon). The intensity of the HCNT peak was determined by fitting the C-1s region collected at an electron emission angle of 0° using a Shirley background and a Doniach-Sunjic line-shape for the helical HCNT. This is based upon fits of the C-1s of highly ordered pyrolytic graphite (HOPG) [6]. The presence of Cl and Al (Figs. S5-C, D) may be due remnant nanoparticles or residues from the synthesis process.

Raman spectroscopy was performed using a Renishaw micro-Raman spectrometer with a 632 nm excitation laser. Raman data were collected under a 50× long working distance objective at 1.3 mW incident power for HCNT powders on glass slides. The Raman spectra (Fig. S6) show two distinct peaks at ~1334 and ~1592 cm^{-1}, commonly referred to as the D and G peaks in carbon nanomaterials. The G peak is attributed to the in plane stretching of the sp^2 hybridized C-C sigma bond. The D peak is attributed to disorder in the crystal structure which breaks translational symmetry, allowing for breathing of the carbon hexagons in the lattice [7].

Size distribution and zeta potential of the HCNTs was performed using a Zetasizer Nano ZS(Red Badge) ZEN3600 (Malvern Instruments, Paris, France) with the nanotubes dispersed in the dispersal media. Particle size distribution of dispersed samples demonstrated a mean diameter of 532 nm (Fig. S7). The zeta potential of dispersed HCNTs in dispersal media was determined to be -3.04 mV.

Molecular, cytotoxic, and immunological assays

Protein concentrations were determined by the bicinchoninic acid protein assay kit (Pierce Chemical Co., Rockford, IL), using bovine serum albumin (BSA) as a standard. Lactate dehydrogenase levels were determined by the CytoTox 96 non-radioactive cytotoxicity assay (Promega, Madison, WI). KC and MCP-1 concentrations were determined using ELISA kits according to procedures by the manufacturer (R&D Systems, Minneapolis, MN).

HCNT exposure and *P. aeruginosa* infection in mice

Six-week old wild-type CD-1 mice (Charles River Laboratories) were housed in positively ventilated microisolator cages with automatic recirculating water, located in a room with laminar, high efficiency particle accumulation-filtered air. The animals received autoclaved food, water, and bedding. Mice were handled in accordance with approved protocols through the Institutional Animal Care and Use Committee at the University of Illinois at Urbana-Champaign. HCNTs (50 µg in 50 µl of PBS/0.01% Tween-80) was intranasally inoculated into the lungs of isoflurane anaesthetized CD-1 mice either once or twice/week for 3 weeks. Control mice were exposed to 50 µl of PBS/0.01% Tween-80. At designated time points, mice were removed and the lungs were lavaged or collected for histopathology.

For infection models, HCNTs or PBS/0.01% Tween-80 exposed mice were given a single intranasal dose of PAO1 (10^7 cfu in 50 µl) 72 hours following the last HCNT administration [8]. After 24 hours of PAO1 infection, mouse lungs were harvested for bacterial enumeration, histopathology, or lavaged for cell enumeration and for ELISA.

For in vivo phagocytosis enumeration, mice were exposed to HCNTs or dispersal media for 3 weeks as described above. Mice were given a single intranasal dose of PAO1-GFP (1×10^7 cfu in 50 µl) 72 hours after the last HCNT treatment. After 24 hours, leukocytes from the lavage were collected. Leukocytes were concentrated onto glass slides using cytospin and cells were fixed with 1% paraformaldehyde overnight at 4ºC. Macrophages were examined with confocal microscopy as described above. The % of cytoplasm occupied by HCNTs was measured using Adobe Photoshop (San Jose, CA).

For neutrophil depletion, mice were exposed to HCNTs or dispersal media for 3 weeks as described above. 24 hours before infection, mice were given 0.2 mg of the

anti-Ly6g antibody (BioXCell, West Lebanon, NH) intraperitoneally. Mice were given a single intranasal dose of PAO1 (10^4 cfu in 50 µl). After 24 hours, mouse lungs were harvested for bacterial enumeration, histopathology, or lavaged for cell enumeration.

For macrophage depletion, mice were exposed to HCNTs or dispersal media for 3 weeks as described above. Mice were given 5 mg of clodronate liposomes (clodronateliposomes.com) intraperitoneally 72 hours before infection followed by 1 mg of clodronate liposomes intraperitoneally before infection. Mice were given a single intranasal dose of PAO1 (1×10^7 cfu in 50 µl). After 24 hours of PAO1 infection, mouse lungs were harvested for bacterial enumeration.

Confirmation of the efficacy of neutrophil and macrophage depletion was accomplished by repeating the depletion and infection methods listed above, analyzing the systemic leukocyte population from blood smears collected by abdominal venipuncture, and analyzing the BAL leukocyte population following cytospin concentration.

Bronchoalveolar lavage (BAL)

BAL of mouse lungs was performed as described [9,10]. The trachea was exposed and intubated with an 18 gauge needle. Four 1 ml aliquots of cold PBS were instilled. The first aliquot was centrifuged at 500 g for 5 minutes at 4ºC and the supernatant was removed and stored separately at -80ºC for ELISA. Cells from the first aliquot were then pooled with the remaining aliquots. Cell numbers were counted by hemocytometer and cell differential was determined microscopically following cytospin

preparation of cells stained with Diff-Quik stain. At least 200 cells per slide were counted.

Histopathology evaluation of mouse lung tissues

Mouse lungs were collected for histopathological analyses as described [11]. Mouse lungs were fixed in 10% neutral buffered formalin, embedded in paraffin, and sectioned and stained with hematoxylin and eosin (H&E) or with Alcian blue. Tissue sections were examined by a pathologist at the Department of Pathobiology, University of Illinois at Urbana-Champaign.

Statistical analysis

Normality of the data was evaluated using the Anderson-Darling normality test with rejection of normality when p-value < 0.05. Data were then analyzed for statistical significance by Student's t-tests, with differences between means considered significant when p-value < 0.05. For comparing the means of groups of three or more, data were analyzed for statistical significance by ANOVA followed by Tukey's tests for comparison between the means.

Results

Cytologic evaluation of leukocyte cytospin preps from BAL of mice collected 24 hours after exposure to HCNTs shows 71.5% of macrophages have phagocytized HCNTs after one exposure and 88% of macrophages have phagocytized HCNTs after 3 weeks of exposure (Figure S8).

References

1. Nečas D, Klapetek P (2012) Gwyddion: An open-source software for SPM data analysis. Central European Journal of Physics 10: 181-188.

2. Meijering E, Jacob M, Sarria JF, Steiner P, Hirling H, et al. (2004) Design and validation of a tool for neurite tracing and analysis in fluorescence microscopy images. Cytometry Part A 58A: 167-176.

3. Harris PJF (2009) Carbon nanotube science: Synthesis, properties, and applications. Cambridge: Cambridge University Press. 130-132 p.

4. Saito R, Dresselhaus G, and Dresselhaus M (1998) Physical properties of carbon nanotubes. London: Imperial College Press. 1-70 p.

5. Peigney A, Laurent C, Flahaut E, Bacsa RR, Rousset A (2001) Specific surface area of carbon nanotubes and bundles of carbon nanotubes. Carbon 39: 507-514.

6. Unarunotai S, Koepke JC, Tsai C, Du F, Chialvo CE, et al. (2010) Layer-by-layer transfer of multiple, large area sheets of graphene grown in multilayer stacks on a single SiC wafer. ACS Nano 4: 5591-5598.

7. Dresselhaus MS, Jorio A, Hofmann M, Dresselhaus G, Saito R (2010) Perspectives on carbon nanotubes and graphene raman spectroscopy. Nano Lett 10: 751-758.

8. Shvedova AA, Fabisiak JP, Kisin ER, Murray AR, Roberts JR, et al. (2008) Sequential exposure to carbon nanotubes and bacteria enhances pulmonary inflammation and infectivity. Am J Respir Cell Mol Biol 38: 579-590.

9. Kuang Z, Hao Y, Walling BE, Jeffries JL, Ohman DE, et al. (2011) *Pseudomonas aeruginosa* elastase provides an escape from phagocytosis by degrading the pulmonary surfactant protein-A. PLoS ONE 6: e27091.

10. Zhang S, McCormack FX, Levesque RC, O'Toole GA, Lau GW (2007) The flagellum of *Pseudomonas aeruginosa* is required for resistance to clearance by surfactant protein A. PLoS ONE 2: e564.

11. Hao Y, Kuang Z, Walling BE, Bhatia S, Sivaguru M, et al. (2012) *Pseudomonas aeruginosa* pyocyanin causes airway goblet cell hyperplasia and metaplasia and mucus hypersecretion by inactivating the transcriptional factor FoxA2. Cell Microbiol 14: 401-415.
